# Antibacterial potential of Propolis: molecular docking, simulation and toxicity analysis

**DOI:** 10.1186/s13568-024-01741-0

**Published:** 2024-07-16

**Authors:** Shabana Islam, Erum Akbar Hussain, Shahida Shujaat, Muhammad Umer Khan, Qurban Ali, Saif Ul Malook, Daoud Ali

**Affiliations:** 1 Department of Chemistry, Lahore College for Women University, Lahore, Pakistan; 2 Institute of Molecular Biology and Biotechnology, The University of Lahore, Lahore, Pakistan; 3 Department of Plant Breeding and Genetics, Faculty of Agricultural Sciences, University of the Punjab, P.O BOX. 54590, Lahore, Pakistan; 4 Department of Entomology and Nematology, University of Florida, Gainesville, USA; 5 Department of Zoology, College of Science, King Saud University, PO Box 2455, Riyadh, 11451 Saudi Arabia

**Keywords:** Propolis, Toxicity, Bacterial protein, Molecular docking, Simulation, ADME

## Abstract

The issue of antibiotic resistance in pathogenic microbes is a global concern. This study was aimed to explore *in silico* and in vitro analysis of the antibacterial efficacy of different natural ligands against bacterial activity. The ligands included in the study were Propolis Neoflavanoide 1, Carvacrol, Cinnamaldehyde, Thymol, p-benzoquinone, and Ciprofloxacin (standard drug S*). The outcomes of molecular docking revealed that Propolis Neoflavaniode-1 showed a highly significant binding energy of − 7.1 and − 7.2 kcal/mol for the two gram-positive bacteria, as compared to the gram-negative bacteria. All ligands demonstrated acute toxicity (oral, dermal), except for Propolis Neoflavanoide 1 and S* drugs, with a confidence score range of 50–60%. Using a molecular dynamic simulation approach, we investigated Propolis Neoflavaniode-1’s potential for therapeutic use in more detail. An MD simulation lasting 100 ns was performed using the Desmond Simulation software to examine the conformational stability and steady state of Propolis Neoflavaniode-1 in protein molecule complexes. Additionally, in vitro studies confirmed the antimicrobial activity of Propolis Neoflavaniode 1 by increasing the zone of inhibition against Gram-positive bacteria, *p* < 0.005 as compared to gram-negative bacteria. This study revealed the promising antibacterial efficacy of Propolis Neoflavaniode 1, demonstrated through robust *in silico* analyses, minimal toxicity, and confirmed in vitro antimicrobial activity, suggesting its potential as a viable alternative to combat antibiotic resistance.

**Supplementary Information:**

The online version contains supplementary material available at 10.1186/s13568-024-01741-0.

## Introduction

Certainly, a population’s health problems and diseases can be caused by a diverse array of bacteria. These microorganisms have a wide range of disease-causing potential, which highlights their significant impact on human health, including issues such as foodborne illnesses, diarrhea, skin infections, respiratory tract infections, and fever (Gonzalez-Martin 2019; Janik et al. 2020). Foodborne infections were a rising public health concern that impedes socioeconomic growth and greatly increases the worldwide burden of illness, death, and disability. Foodborne infections can arise at any step of the food’s manufacture, processing, distribution, or consumption (AMJAD et al. 2018; Ishaq et al. 2021; Ullah et al. 2023a, b).

AMR, or antimicrobial resistance, first appeared in the 1940s, not long after penicillin became widely used. Since then, AMR has developed into a significant danger to world health. It was estimated that AMR causes 700,000 deaths yearly, which were expected to rise to 10 million deaths by 2050. The economic cost of AMR was also significant, estimated to be $100 billion per year. According to World Bank estimates, AMR could result in yearly GDP losses of 3.8% by 2050, causing global economic harm comparable to the shocks seen during the 2008 financial crisis (Shevade and Naik 2023).

Bacteria that primarily affect the gastrointestinal (GI) tract and were classified as enteric bacterial pathogens can cause a range of gastrointestinal diseases (Axelrad et al. 2021). They were frequently spread through contaminated food, drink, or contact with infected individuals. Some common intestinal bacterial pathogens include *Salmonella enterica* serotypes *Typhimurium* and Enteritidis, which were responsible for causing salmonellosis and symptoms such as diarrhea, stomach pains, nausea, vomiting, and fever. ETEC and EHEC were also significant intestinal bacterial pathogens. ETEC was known to cause traveler’s diarrhea, while EHEC, such as O157:H7, can lead to severe foodborne illness with symptoms like bloody diarrhea and hemolytic uremic syndrome (Roussel et al. 2017; Rojas-Lopez et al. 2018; IQBAL et al. 2021; Ullah et al. 2023a, b).

Likewise, bacterial, viral, and other microbial infections can all cause respiratory tract infections. Specific bacterial pathogens, including *Staphylococcus aureus*,* Streptococcus pneumonia*, and *hemophilic influenza*, were commonly responsible for bacterial respiratory infections (Wu et al. 2021; GOHAR et al. 2023; Hassan et al. 2023). *Hemophilic influenza* is not only a primary cause of pneumonia but can also lead to bronchitis and sinusitis (Diaz-Diaz et al. 2022).

Antimicrobials have been used extensively recently to treat infections both therapeutically and preventatively. When bacteria, viruses, fungi, or parasites adapt and create defense mechanisms against antimicrobial drugs, it has been known as antimicrobial resistance (Schwartz and Morris 2018; AMIN et al. 2023; Bhatti et al. 2023; Upadhayay et al. 2023). As a result, standard treatments become ineffective, and resistant bacteria can persist and spread. Methicillin-resistant Staphylococcus aureus (MRSA), Klebsiella pneumonia, and 50% of Escherichia coli were resistant to third-generation cephalosporins and fluoroquinolones. The mortality rate for cultured multidrug-resistant (MDR) strains varies greatly, ranging from 5 to 17% in European countries and an alarming 46–67% in Asian countries. In light of these statistics, it was vital to explore alternative therapeutic approaches to combat the growing threat of AMR, in addition to relying on conventional antibiotics (Chaudhary 2016).

While a wide range of clinical illnesses can be treated with plants, only 15% of them have been studied phytochemicals, and only 6% have undergone biological activity screening (Barbălată-Mândru et al. 2022). The rise in antibiotic resistance brought on by the extensive use of antibiotics is another problem impacting public health. Community-acquired pathogens include bacteria such as *Staphylococcus*,* Shigella*, *Enterococcus sp*., and *Escherichia coli*, which were among the most prevalent bacteria with multidrug resistance. Coagulase-negative bacteria, *Salmonella species*,* and Pseudomonas aeruginosa* were also included in this category. Because of this, patients now vehemently desire new antibiotics to treat infections, and researchers have become increasingly intrigued by the potential antibacterial properties of herbal therapies (Hannan et al. 2014). Because of their antifungal, antibacterial, and antiviral properties, plant extracts and essential oils have been studied extensively around the world for their potential as new sources of antimicrobial chemicals, food preservation agents, and alternative treatments for a variety of ailments (Chouhan et al. 2017; Puvača et al. 2021).

Bees gather Propolis, a resinous compound, from a variety of plants and utilize it as a structural material, cleaner, and sealant inside the hive. Its possible health benefits—including its antimicrobial qualities—have drawn attention. Propolis contains a subclass of flavonoids called Neoflavonoids, which were thought to be involved in some of the plant’s biological actions. Neoflavonoids found in Propolis have the direct ability to prevent bacterial development and multiplication. They could damage the membranes surrounding bacterial cells, obstruct cellular metabolism, or suppress vital enzymes needed for bacterial survival (Okińczyc et al. 2020).

Computer-aided drug design (CADD) is a potent tool in the drug discovery space that finds, optimizes, and develops novel drug candidates through computational techniques and simulations. Prioritizing and filtering compounds using CADD helps researchers reduce the number of molecules that require experimental synthesis. This decrease in the quantity of synthesized molecules can result in significant savings in labor, reagent, and equipment costs. The computational technique known as “molecular docking” was used in structural biology and drug development to anticipate the binding mechanism and affinity of a small molecule (ligand) with a target macromolecule (Abbas et al. 2021; Nascimento et al. 2022). Antimicrobials have been used as both preventative and treatment to combat infections. However, the excessive use of antimicrobials, especially antibiotics, has led to a concerning rise in antimicrobial resistance (AMR) worldwide.

The objective of the present study was to study Propolis Neoflavaniode 1, Carvacrol, Cinamald ehyde, Thymol, and p-benzoquinone wae among the chemicals that were found. Their ligand binding affinities to gram-positive and gram-negative bacteria were assessed using an *in silico* toxicity analysis and *in silico* binding docking technique. Later, the in vitro antibacterial effectiveness of these compounds was tested to validate the *in silico* technique.

## Materials and methods

### Dataset of target proteins and preparation

*Bacillus subtilis and Staphylococcus aureus* are examples of gram-positive bacteria whose protein molecules were chosen from the Protein Data Bank (PDB). Specifically, the membrane protein Bmr (PDB ID: 1BOW) and Pencillin binding protein-1 (PDB ID: 5TR0) were retrieved from the PBD database. Protein molecules from gram-negative bacteria, including dehydratase (PDB ID: 1U1Z), dispersin (PDB ID: 2JVU), and ompC (PDB ID: 3UU2), were obtained from the PDB database. The bacteria were *Escherichia coli*,* Salmonella typhimurium*,* and pseudomonas aeruginosa*. This study examined the interactions between several natural chemicals and known bacterial protein molecules to assess the potential antibacterial activity of each. The MOE program received the exported protein molecule (Berman et al. 2000).

### Dataset of ligands and preparation

A set of ligands the structure, PubChem compound identity number, Thymol (CID: 6989), Carvacrol (CID: 10,364), Cinnamaldhyde (CID: 637,511), Propolis Neoflavonoide 1 (CID: 11,148,893), p-benzoquinone (CID: 4650), and standard drug Ciprofloxacin (CID: 264). The structures of the compounds were downloaded in SDF format from PubChem. The ligands were exported into the MOE software (Bolton et al. 2011).

### Molecular docking of natural compounds

Molecular docking was a process of investigating the interactions between protein and ligand molecules to identify the most effective method of bonding and creating a stable complex. In this study, the MOE software was utilized to dock and assess the docking score, structural interactions, and stability of gram negative and gram positive bacterial receptors with five natural chemicals, as well as compare them to the standard drug Ciprofloxacin (S*). The process of visualizing the docked model complex involved using MOE tools, and the most precise structure was selected based on the docking score or binding energy. Using the protein-ligand docking approach, the binding conformation of putative inhibitors inside the binding region of bacterial receptors was shown. The binding energy of MOE was quantified in kcal/mol through the use of the S-score. The force fields in the scoring function were used to adjust the docking results using the GBVI/WSA dG scoring approach, which has been based on the generalized Born solvation model. When docking, the ligand was thought to be flexible and the receptor to be static. Using a point selection technique, the x, y, and z coordinates of an estimated centroid point were determined. The radius value of the area containing all active site residues around the centroid was determined. A maximum of 1000 postures were attempted with each ligand, and the long docking procedure was selected because of its exceptional precision. The resulting postures, which included ligand internal and exterior protein-ligand interaction energies, were ranked using the fitness function in the S-score (ULC, 2020).

### Lipinski rule analysis using Molinspiration

The analysis of drug-like properties for natural antibacterial compounds was conducted using the Molinspiration (Bai et al. 2022) which requires input in the form of compound smiles. These online server was employed to predict the molecular properties and bioactivity of compounds exhibiting high affinity. The tool calculates descriptors, including logP, polar surface area, mass, range of atoms, range of O or N, range of OH, range of rotatable bonds, volume, and drug likeness.

### *In silico *absorption, distribution, metabolism, excretion, and toxicity (ADMET) analysis

Swiss ADMET server was employed to assess the physicochemical and pharmacokinetic properties of the identified compounds. Additionally, the toxicity properties of these compounds were analyzed using the online servers Protox-II and StopTox, which necessitate input in the form of compound smiles (Daina et al. 2017).

### MD simulation trajectory

Prior to MD simulation, protein and ligand analysis was carried out as a crucial initial step in predicting a static view of the molecule’s binding position at the protein’s active site (Ferreira et al. 2015). Newton’s classical equation of motion was frequently included in MD simulations to replicate atom movements over time and predict the state of ligand binding in a physiological situation. The ligand-receptor combination was optimized, minimized, and any missing residues were filled in using Maestro’s Protein Preparation Wizard. The System Builder tool was used to build the system. Utilizing an orthorhombic box with an OPLS_2005 force field, 300 K temperature, and 1 atm pressure, the solvent model TIP3P (Intermolecular Interaction Potential 3 Points Transferable) was used. 0.15 M sodium chloride was used to neutralize the models and imitate physiological circumstances, respectively. The models were relaxed before simulation began, and trajectories were saved for safety assessment every 100 ns (Hildebrand et al. 2019).

### Dynamic cross correlation matrix and principle component analysis (PCA) of protein-ligand complexes

Dynamic cross correlation matrix (DCCM) was constructed across all Cα-atoms for all complexes during the 100 ns MD simulation in order to investigate the domain correlations. The global motions of the trajectories during the 100 ns simulation of the PBP-1 complexes and membrane protein Bmr with Propolis Neoflavanoide 1 were using PCA analysis. To compute PCA, a covariance matrix was created as previously said. The various principal component conformational modes were computed as the trajectories moved, and a comparison of the initial highest mode was examined for the Propolis Neoflavanoide 1 bound complex’s conformational analysis. The Propolis Neoflavanoide 1 bound complex’s free energy landscape for protein folding was assessed. We convert trajectories into DCD file and run R script by using bio 3d package (Grant et al. 2006).

### Preparation of sample solutions

Forty Propolis samples were collected from different regions of Punjab, Pakistan. The samples were divided into four groups (*I-VI*), 10 in each group and carefully transported in dark bags. They were kept at − 20 °C until further used. The frozen samples were crushed and dissolved in 70% ethanol (1:10). Solutions were macerated in dark for one week and filtered. Filtrates were kept overnight in refrigerator to dewax. Wax was removed and clear samples were used for antibacterial assay.

### Antibacterial activity of Propolis Neoflavaniode 1 compound

#### Preparation of nutrient broth

The process of creating the agar medium involved dissolving 28 g of nutrient agar in 1000 milliliters of distilled water in a conical flask. After that, the flask was autoclaved for 15 min at 121 degrees Celsius and 15 pounds of pressure (Ganeshamurthy et al. 2021).

#### Preparation of bacterial culture medium

Five distinct bacterial strains were added individually to each flask under a laminar flow hood after the culture medium had been autoclaved. Three gram negative bacteria (*Pseudomonas aeruginosa* (ATCC 35,984), *Escherichia coli* (ATCC 25,922), and *Salmonella typhimurium* (ATCC 14,028) and two gram positive strains (*Bacillus Subtilis* (ATCC 6051) and *Staphylococcus aureus* (ATCC 6538) were combined into two divisions. The flasks were covered and kept overnight at 37 degrees Celsius in a shaking incubator. When the flasks were removed from the incubator after a full day, the turbidity in the medium—a sign of bacterial growth was seen (Bonnet et al. 2020; Wang et al. 2021).

#### Preparation of agar medium

The process of creating the agar medium involved dissolving 28 g of nutrient agar in 1000 milliliters of distilled water in a conical flask. Then for 15 min, the flask was autoclaved at 121 degrees Celsius and 15 pounds of pressure (Das et al. 2023).

#### Preparation of agar plates and sample loading

Agar medium (25 mL) was prepared and placed into sterilized petri dishes within a laminar flow hood. These petri dishes were then allowed to cool. Next, 0.5 MacFarland scale microbe suspensions were added to the solid medium plates. Using a cork borer, wells were created, and 20 µl of the sample was placed into each well. Ethanol served as the control, while a 20 µl Ciprofloxacin antibiotic solution was used as the standard. After that, the plates were incubated at 37 ºC for 24 h and inhibitory zone diameters were then measured in ml (Fadakar Sarkandi et al. 2021). Each experiment was performed in triplicate.

### Broth dilution assay (MIC)

The macro broth dilution method was utilized to determine the minimum inhibitory concentration (MIC) for every strain of bacteria. A known amount of tested strains (105 CFU/mL) and propolis extract in serial concentrations: 0 (negative control), 0.01, 0.02, 0.03, 0.05, 0.06, 0.07, 0.08, 0.09 mg/mL were added to each well of a 48-well plate. The turbidity was utilized to calculate the minimal inhibitory concentration following a 24-hour incubation period at 37 °C. In order to ensure sufficient bacterial growth, broth and inoculum were added to provide a negative control (Belanger and Hancock 2021).

### Statistical analysis

Applied the Shapiro-Wilk test to assess the normality distribution of the data. As the data was non-parametric, we used the Kruskal-Wallis test for group comparisons. Clarified the descriptive analysis for MIC and included p-values for comparisons to highlight the significance levels.

## Results

### Molecular docking

The most effective method for identifying protein-ligand interactions in molecular models was graded by using the MOE grading system. The binding score were evaluated and the docked pose was confirmed inside the binding pocket.

To compare our chosen natural compounds, including Thymol, Carvacrol, Cinamaldehyde, Propolis Neoflavonoide-1, p-benzoquinone and the standard drug (S*) against the bacterial protein motif acting as a receptor, the findings show that the compounds have strong binding affinities to particular bacterial outer membrane proteins, indicating that they may be able to inhibit bacterial activity. In terms of overall energy, the optimal binding energy was represented by the lowest value.

In our study, we aimed to investigate the interactions between various ligands and G-positive bacterial protein motifs. For this purpose, we selected *Bacillus cerus* (PDB ID: 2NYP) and *Staphylococcus aureus* (PDB ID: 1TVF). The interaction between Propolis Neoflavanoide 1 and crucial residues of membrane protein Bmr and PBP-1 molecule was found to have the lowest binding energy at − 7.2 and − 7.0 kcal/mol. Analysis of the protein-ligand interface was conducted, and Propolis Neoflavanoide 1 compound complex with these residues (ASP-90, HIS-149, Lys-606, Asp-516) included bond distances of 2.72 Å, 2.82 Å, 2.67 Å, and 3.1 Å. The residues were significant as they form two hydrogen bonds and were located inside the binding pocket. The P Propolis Neoflavanoide 1 also showed the highest binding energy against the gram-positive bacterial protein motif. MOE scores for the Propolis Neoflavanoide 1 compound docking against three gram-negative bacterial protein molecule (3R)-hydroxymyristoyl-[acyl carrier protein Dehydratase, ompC, and Dispersin were − 5.8 kcal/mol, − 6.3 kcal/mol, and − 6.9 kcal/mol, respectively. According to the 2D diagram, Glu-63 of Dehydratase, a key amino acid for interaction with Propolis Neoflavanoide 1 and forms one hydrogen bond with a distance of 2.3 Å. Ser-322 and Gln-356 also interact with Propolis Neoflavanoide 1 near the binding pocket of ompC, forming two hydrogen bonds with distances of 2.6 Å and 2.3 Å, respectively, indicating strong potential for inhibitory interactions that could stop the activity of the binding site, see in (supplementary Fig. 1(a-j)) and Table 1.

**Table 1:** Molecular docking of Gram-positive and Gram-negative bacterial protein with different ligands and standard drug (S*)

		Gram-positive bacteria		Gram-negative bacteria		
**Ligands**		Bmr	PBP-1	Dehydrates	ompC	Dispersin
Propolis Neoflavonoide 1	S-score	-7.2 kcal/mol	-7.0 kcal/mol	-5.8 kcal/mol	-6.3 kcal/mol	-6.9 kcal/mol
	Direct interacting residue	ASP-90, HIS-149	Lys-606, Asp-516	Glu-63	Ser-322 and Gln-356	Asp-152
	H-bond	2	2	1	2	1
Carvacrol	S-score	-6.2 kcal/mol	-6.1 kcal/mol	-6.1 kcal/mol,	-6.0 kcal/mol	-6.1 kcal/mol
	Direct interacting residue	Glu-63	Ala-110	Ser-29	Glu-63	Asp-90
	H-bond	1	1	1	1	2
Cinamaldhyde	S-score	-6.2 kcal/mol	-6.2 kcal/mol	-6.0 kcal/mols	-6.0 kcal/mol	-5.6 kcal/mol
	Direct interacting residue	Thr-444	Tyr-88	Leu-115, Lys-114	Thr-444	Gly-179, Lys-171
	H-bond	1	1	2	1	1
Thymol	S-score	-6.4 kcal/mol	-5.8 kcal/mol	-5.6 kcal/mol	-5.9 kcal/mol	-6.3 kcal/mol
	Direct interacting residue	Gln-63	Leu-178	Ala-110	Gln-521	Glu-63
	H-bond	1	1	1	1	1
p-benzoquinone	S-score	-5.9 kcal/mol	5.7 kcal/mol	-6.0 kcal/mol	-6.1 kcal/mol	-5.9 kcal/mol
	Direct interacting residue	His- 149, His-86	Asn-464, Gln-521, Lys-430	Tyr-86	Lys-16	Nill
	H-bond	2	3	1	1	0
Ciprofloxacin standard drug (S*)	S-score	-6.4 kcalmol	-6.9 kcal/mol	-6.2 kcal/mol	-7.1 kcal/mol	-7.0 kcal/mol
	Direct interacting residue	Tyr-87	Asp-105, Arg-124, Tyr-115	Lys-63, Ile-88, Arg-136	Gln-521	Lys-176, Asp-177, Asp-90
	H-bond	1	3	3	1	3

According to our results, Carvacrol showed the least binding energy with membrane protein Bmr and Penicillin binding protein 1, at − 6.2 and − 6.1 kcal/mol, respectively. The 2D graph indicates one H-bond formation between Thyr-444 and Lys-11 with a bond distance of − 2.61 and − 2.79 Å. In the case of negative bacterial protein motifs, dehydratase, ompC, and Dispersin had the least binding energy at − 6.1, − 6.0, and − 6.1 kcal/mol, respectively. The 2D graph shows three H-bond formation between Glu-63, Ser-29, and Ala-110 with bond distances of 2.58, 2.82, and 2.7 Å. These findings suggest that Carvacrol has shown highest binding energy in both gram-positive and gram-negative bacteria, as shown in (supplementary Fig. 2(a-j)) and Table 1.

Our findings revealed that Cinamaldehyde exhibited the least binding energy of − 6.2 and − 6.2 kcal/mol with membrane protein Bmr and PBP-1, respectively. 2D graph interpretation of the complex between the ligand and protein showed two H-bond formations involving protein residues Gln-63 and Leu-178 with bond distances of − 2.61 and − 2.79 Å. For the negative bacterial protein molecules, including dehydratase, ompC, and dispersin, displayed the lowest binding energy of respectively, as shown in (supplementary Fig. 3(a-j)) and Table 1.

Our findings revealed that Thymol exhibited the least binding energy of − 6.4 and − 5.8–6.2 and − 6.2 kcal/mol with membrane protein Bmr and PBP-1, respectively. 2D graph interpretation of the complex between the ligand and protein showed two H-bond formations involving protein residues Thr-444, Tyr-88 with bond distances of 2.2 and 2.1 Å. For the negative bacterial protein motifs, including dehydratase, ompC, and dispersin, Thymol displayed the lowest binding energy of − 5.6 kcal/mol, − 5.9 kcal/mol, and − 6.3 kcal/mol, respectively. Additionally, 2D graph interpretations of the complexes between the ligand and protein showed two H-bond formations involving Ala-110, Gln-521, and Glu-63 with bond distances of 2.58, 2.82, and 2.7 Å. These results suggest that Thymol has potential for highest binding energy in both gram-positive and gram-negative bacteria, as shown in (supplementary Fig. 4 (a-j)) and Table 1.

The interactions between the p-benzoquinone and crucial residues of the membrane protein Bmr and PBP-1 were found to have the least binding energy of − 5.9 and − 5.7 kcal/mol, as determined through protein-ligand interface analyses. According to the 2D diagram, His-149 and His-86 were key amino acids that contribute to forming two hydrogen bonds with PBP2a, with 2.75 Å and 3.01 Å, respectively. Asn-464, Gln-521, and Lys-430 have also been shown to interact and form three hydrogen bonds with the p-benzoquinone, located close to the binding pocket, with bond distances of 2.88 Å, 2.7 Å, and 2.76 Å, respectively. The p-benzoquinone displayed the lowest binding energy against gram-positive bacterial protein receptors, as shown in (supplementary Fig. 5 (a-j) and Table 1.

Conversely, the reference drug Ciprofloxacin exhibited the least binding energy of − 7.2 and − 7.0 kcal/mol when interacting with the gram-positive target molecule of membrane protein Bmr and PBP-1, as determined through protein-ligand interface analyses. The standard drug Ciprofloxacin complex with beta lactamase II and PBP-1 residues, including Tyr-87, Asp-105, Arg-124, Tyr-115, with bond distances of 2.72 Å, 2.82 Å, 2.67 Å, 3.1 Å, respectively, were considered significant residues as they form strong hydrogen bonds and were located within the binding pocket. The standard drug Ciprofloxacin and G-negative bacterial protein molecule residues, such as Dehydratase, ompC, and Dispersin, exhibited the least binding energy of − 6.2, − 7.1, and − 7.0 kcal/moil, respectively, with residues Lys-63, Ile-88, Arg-136, Gln-521, Lys-176, Asp-177, and Asp-90 forming strong hydrogen bonds with target site. Interestingly, Ciprofloxacin was found to display the highest binding energy, similar to the Propolis Neoflavanoide 1 compound, as shown in (supplementary Fig. 6 (a-j)) and Table 1.

Our results indicate that Propolis Neoflavanoide 1 displays superior binding performance with respect to membrane protein Bmr and PBP-1 when compared to other compounds. These findings imply that propolis was a more effective antimicrobial agent than Thymol, Carvacrol, Cinnamaldehyde, and p-benzoquinone.

### Lipinski rule analysis using Molinspiration

Molinspiration was used to compute the attributes of the five natural antibacterial compounds, including their LogP, TPSA, Mol. Wt., and the number of hydrogen bond acceptor (nON) and donor (nOHNH). These compounds satisfied the requirements of Lipinski’s rule of five, which stipulates that an oral medication should not break more than one of the following rules: molecular weight < 500Da, hydrogen bond donor < 5, WLogP < 5, and hydrogen bond acceptor < 10. The logP value between 1 and 3 has been typically considered ideal for the target compounds, as it avoids being overly lipophilic while still having enough hydrophobicity for acceptable membrane permeability. The ideal logP value for drug-like substances such as Propolis Neoflavanoide 1, Cinamaldehyde, and p-benzoquinone has been often considered to be between 1 and 3. This range allows for sufficient hydrophobicity to ensure acceptable membrane permeability while avoiding issues such as poor water solubility or non-specific binding. Our target compounds, including thymol, Carvacrol, cinamaldehyde, Propolis Neoflavanoide 1, and p-benzoquinone, all fall within show good (LogP < 5), and (logP) except for Ciprofloxan.

Regarding the TPSA values, it has been often observed that compounds with lower TPSA values (< 140 Å²) have greater oral bioavailability due to their drug-likeness and bioavailability. All of the target compounds were within this range. Additionally, lower TPSA values (< 140 Å²) were often associated with greater oral bioavailability, as smaller, less polar molecules are more easily absorbed by the digestive system. All of the natural compounds and reference drugs followed Lipinski’s rule.

Smaller, less polar molecules were typically more readily absorbed by the digestive system. Conversely, drugs targeting tissues tend to have TPSA values > 140 Å² and may experience incomplete oral absorption, limited cell membrane permeability, and poor absorption. All natural compounds were evaluated in accordance with the Lipinski rule, as shown in (Table 1).

### ADMET analysis

Swiss ADME software was used to analyze the physicochemical characteristics, water solubility, pharmacokinetics, and drug-like qualities of the natural substances. According to the findings, every natural ligand that was evaluated exhibited medication similarity without going against Lipinski’s five-rule. With a bioavailability score of 0.55 for every natural chemical, there was a possibility that these substances could function as drugs and can influence certain tissues. It has been crucial to remember that chemical drug ability prediction does not provide conclusive proof of a compound’s superiority over other molecules. It has been, instead, an in silico approximation of the molecule’s probability of turning into a medication. It was discovered that the chemicals under investigation could pass through the blood-brain barrier and had a high rate of absorption in the gastrointestinal tract. None of the substances under investigation interacted with P-gp, a transmembrane efflux pump that removes foreign substances—including medications—from cells, causing further metabolic, clearance, and pharmacokinetic effects. Furthermore, none of the examined phytochemicals interacted with CYP450, demonstrating their non-toxicity and efficacy. Thymol, Carvacrol, Cinamaldehyde, Propolis Neoflavanoide-1, and p-benzoquinone were similarly shown to have excellent passive gastrointestinal absorption in humans, indicating that they had good oral bioavailability comparable to that of the reference medication. These findings collectively imply that the studied natural chemicals have a good chance of reaching therapeutic dosages at the intended locations.

Therapeutic drug metabolism was facilitated by the CYP450 enzyme family, which contains enzymes that metabolize xenobiotics. This family of enzymes has been found essential for medication clearance in the liver. Since small compounds frequently result in drug-drug interactions that were connected to pharmacokinetics, their inhibition of CYP450 was an important consideration when evaluating their toxicity profiles. It’s crucial to remember that several substances do not inhibit CYP2C9, including p-benzoquinone, thymol, carvacrol, cinamaldehyde, and propolis neoflavanoide 1 in Table 2.

**Table 2:** ADMET profile of multiple ligands (MLP) and S* drug

Ligands Name	Chronic Toxicity by Pro Tox II		Pharmacokinetics by Swiss ADME	
**Propolis-Neoflavaniode 1**	Hepatotoxicity	No	P-gp substrate	No
	Carcinogenicity	No	CYP450 inhibitor	No
	Immunotoxicity	No	BBB	Yes
	Mutagenic	No	GI absorption	High
	Cytotoxicity	No		
**Carvacrol**	Hepatotoxicity	No	P-gp substrate	No
	Carcinogenicity	No	CYP450 inhibitor	No
	Immunotoxicity	No	BBB	Yes
	Mutagenic	No	GI absorption	High
	Cytotoxicity	No		
**Cinamaldehyde**	Hepatotoxicity	No	P-gp substrate	No
	Carcinogenicity	No	CYP450 inhibitor	No
	Immunotoxicity	No	BBB	Yes
	Mutagenic	No	GI absorption	High
	Cytotoxicity	No		
**Thymol**	Hepatotoxicity	No	P-gp substrate	No
	Carcinogenicity	No	CYP450 inhibitor	No
	Immunotoxicity	No	BBB	Yes
	Mutagenic	No	GI absorption	High
	Cytotoxicity	No		
**p-benzoquinone**	Hepatotoxicity	No	P-gp substrate	No
	Carcinogenicity	No	CYP450 inhibitor	No
	Immunotoxicity	No	BBB	No
	Mutagenic	No	GI absorption	High
	Cytotoxicity	No		
**Ciprofloxacin**	Hepatotoxicity	No	P-gp substrate	Yes
	Carcinogenicity	No	CYP450 inhibitor	No
	Immunotoxicity	No	BBB	No
	Mutagenic	No	GI absorption	High
	Cytotoxicity	No		

To evaluate the in-silico toxicity properties of these phytochemicals, the Protox-II and StopTox software were used. The results showed that all of the natural compounds exhibited no hepatotoxicity, carcinogenic, mutagenic, or immunotoxin effects using Protox II. Additionally, all of the tested phytochemicals were found to have acute toxicity after inhalation, oral, and dermal exposure, with confidence levels ranging from 50 to 60%, according to the StopToxs software’s liability prediction. Ciprofloxacin and propolis Neoflavanoide 1 showed similar results, with no inhaled, oral, or dermal toxicity and confidence levels ranging from 50 to 60%. On the other hand, the other compounds, such as thymol, Carvacrol, Cinamaldehyde, and p-benzoquinone, showed greater acute toxicity, with confidence levels above 60% in (Table 2).

### MD simulation

**Fig. 1:**
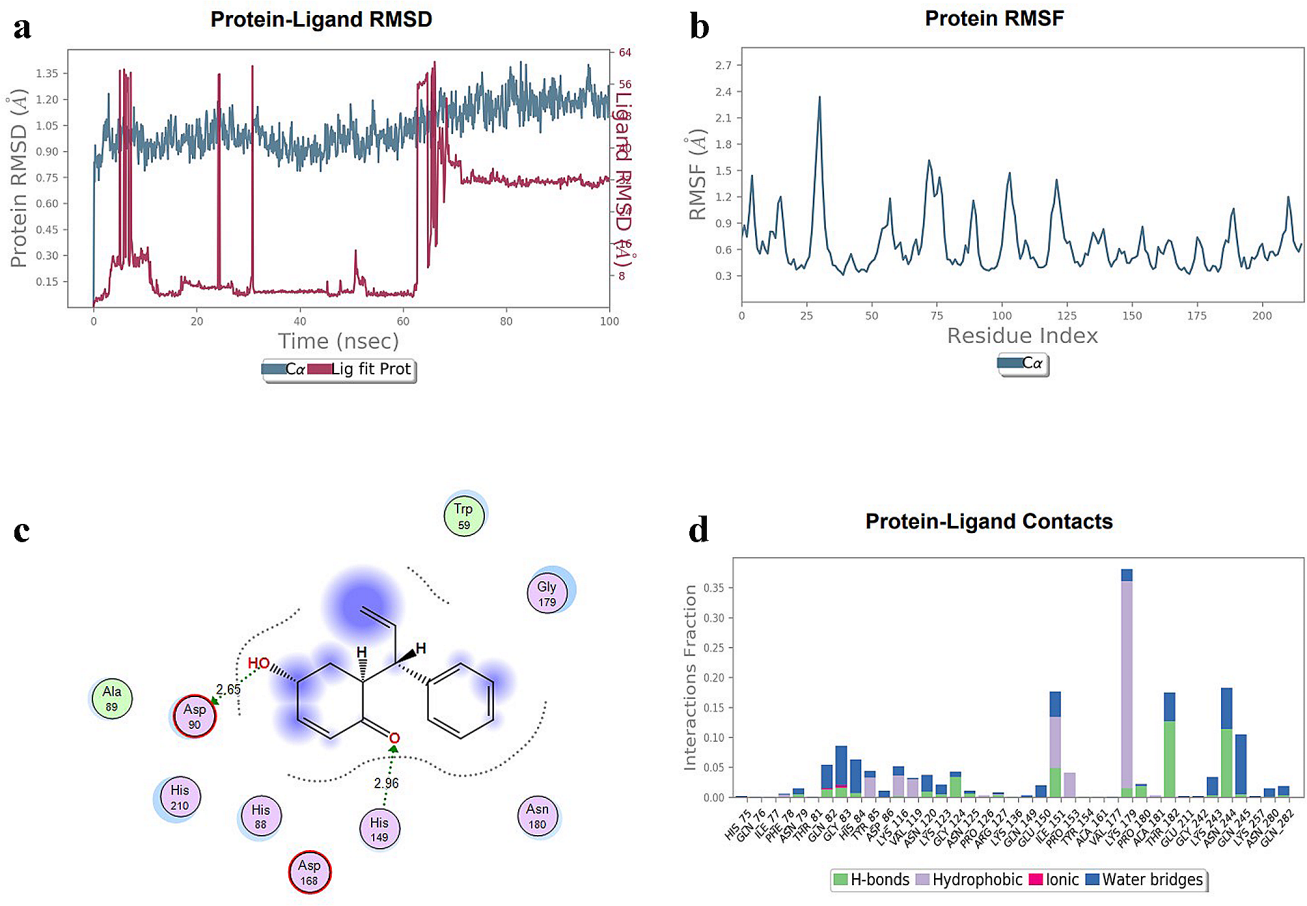
MD simulation analysis of the Propolis Neoflavaniode-1 with membrane protein Bmr complex. (**A**) The residual mean square deviations (RMSDs) of protein Cα atoms and the ligand relative to the protein are displayed in blue and red, respectively. The stability of the complex was maintained as evidenced by the RMSD value of 1.3 Å for the maximum value of membrane protein Bmr Cα atoms. Initially, the RMSD values of the ligand decreased until 18 ns and then stabilized with a mean RMSD of 3 Å. (**B**) The protein RMSFs represent the individual fluctuations of each amino acid during the simulation. (**C**) The 2 D interaction diagram shows the ligand-ligand interaction. (**D**) The protein-ligand contact histogram displays the distribution of contact between the protein and the ligand

Because of its remarkable free-energy interactions with gram-positive bacterial protein molecules, such as those of *Staphylococcus aureus* (PDB ID: 5TR0) and *Bacillus subtilis* (PDB ID: 1BOW), Propolis Neoflavanoide 1 was chosen. Among the metrics that were computed using the MD simulation trajectory were the protein-ligand interaction histogram, RMSD of ligand atoms, RMSD of protein C-α atoms, and protein root mean square fluctuation (RMSF). Based on the reference frame backbone, Fig. 1(a) shows the RMSD of the membrane protein Bmr and Penicillin-binding protein 1 (PBP-1) C-α backbone (light blue hue) during the simulation. The Bmr C-α backbone of the membrane protein had a maximum RMSD of 1.35 Å, suggesting that the Propolis Neoflavanoide 1 complex remained stable during the simulation. The ligand Propolis Neoflavanoide 1’s RMSD in association with membrane protein Bmr fluctuated significantly at first, but after roughly 60 ns of simulation, it stabilized at a consistent RMSD of 1.35 Å. Up to 100 ns, there were only slight changes seen. Throughout the remainder of the simulation, the complexes stayed stable.

During the simulation, the root-mean-square fluctuations (RMSFs) of the protein were compared with the structure of a medicinal drug to determine the mobility of amino acid residues. The protein’s RMSF plot showed sections that were alpha-helical, beta-stranded, and looped. The protein’s beta-strand and alpha-helical sections were less flexible and more rigid than the unstructured loop sections. Little movements were seen in the atoms of the main chain and active site, suggesting only modest conformational alterations. As a result, the cavity of the binding pocket held the reported lead compound. Figure 1(b) displays the RMSF plot of the Propolis Neoflavanoide 1 with Bmr to create complex. Propolis Neoflavanoide 1interacts with amino acids in Bmr membrane protein, including HIS-75, GLN-76, ILE-77, PHE-78, ASN-79, THR-81, GLN-82, GLY-83, HIS-84, TYR-85, ASP-86, LYS-116, VAL-119, ASN-120, LYS-123, GLY-124, ASN-125, PRO-126, ARG-127, LYS-136, GLN-149, GLU-150, ILE-151, PRO-153, TYR-154, ALA-161, VAL-177, LYS-179, PRO-180, ALA-181, THR-182, GLU-211, GLY-242, LYS-243, ASN-244, GLN-245, LYS-257, ASN-280, and GLN-282 as shown in Fig. 1(c).

The green-colored vertical bars indicate all interacting residues. Specifically, GLN-76, ILE-77, TYR-154, ALA-161, and VAL-177 fluctuated highly at 3.5 Å, and remaining amino acids 1.5 Å. The Propolis Neoflavaniode 1 -Bmr complex also engages in hydrophobic interactions, primarily water-mediated bridges, with ASN-47, ASP-168, GLU-141, ASN-195, and ASN-180 residues. In addition, The protein’s crystal structure shows that Propolis Neoflavaniode 1 interacts with THR-183 and ASN-244 through hydrogen bonding and water-mediated interactions. Other interactions were also detected with ASP-90, HIS-149, GLU-141, and ASN-195, but these were persistent throughout the simulation see Fig. 1(d).

**Fig. 2:**
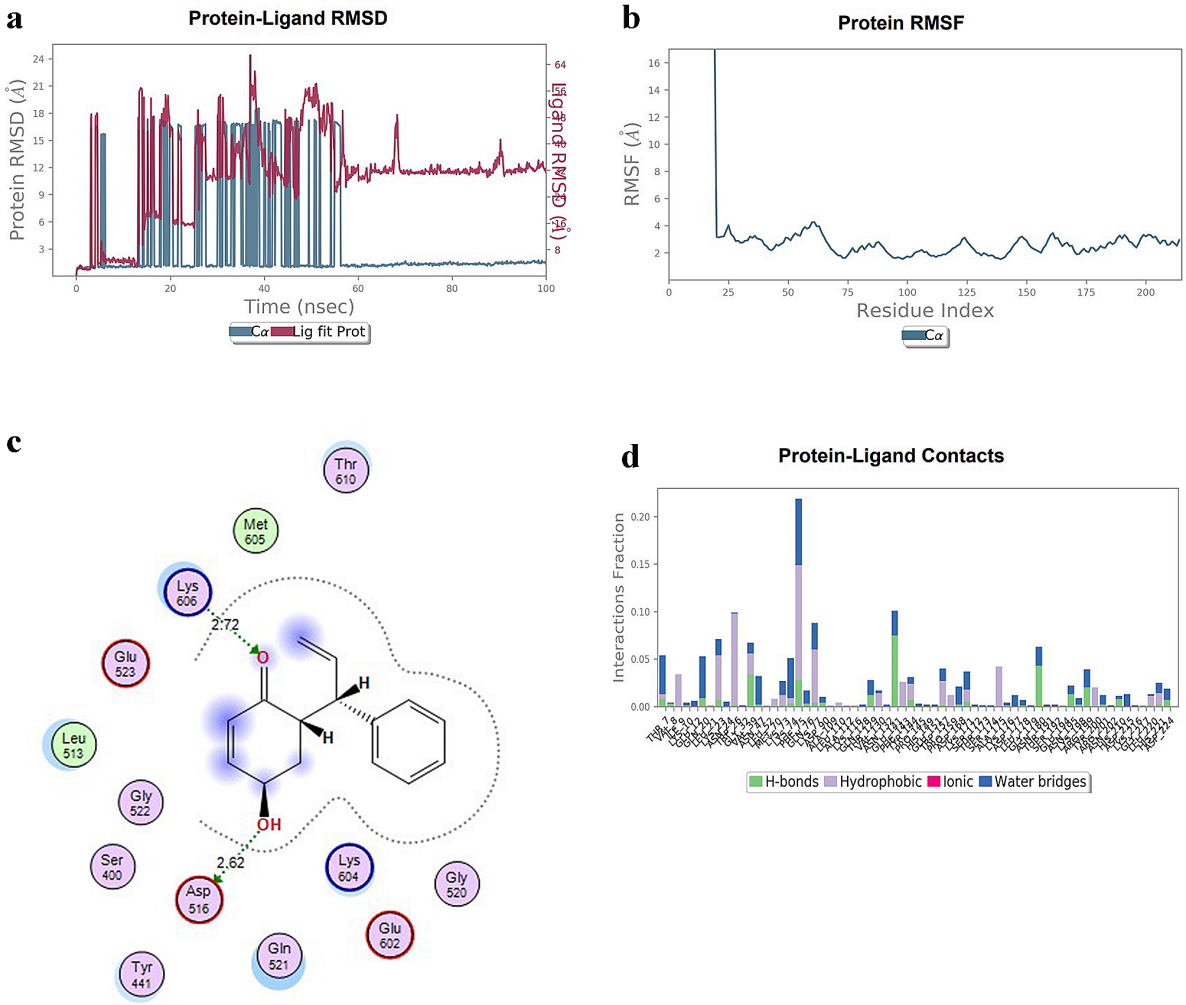
MD simulation analysis of the Propolis Neoflavaniode-1 with PBP-1 complex. (**A**) RMSD of the protein Cα atoms and the ligand relative to the protein is shown over time. The RMSD for the protein is plotted in blue, while the RMSD for the ligand is shown in red. (**B**) The Protein Root Mean Square Fluctuation (RMSF) represents the individual fluctuations of each amino acid during the simulation. (**C**) A diagram depicting the 2D interactions between the ligand and the protein. (**D**) A histogram illustrating the contact points between the protein and the ligand

With an RMSD of 1.35 Å for the PBP-1 C-α backbone, it appears that the Propolis Neoflavanoide 1-PBP complex remained stable over the simulation. When used in conjunction with PBP-1, the ligand Propolis Neoflavaniode 1 fluctuated a lot at first before stabilizing at 55 ns and then fluctuating only little until 100 ns. There were no more variations for the rest of the simulation. Consequently, the pocket cavity of the HPA contained the stated lead compound. According to this plot, Propolis Neoflavanoide 1 interacted with PBP-1’s amino acids, including THR-7, VAL-8, ILE-9, LYS-10, GLU-12, GLN-20, LEU-21, LYS-23, ASN-24, TRP-26, GLT-32, VAL-39, ASN-4, LEU-52, MET-70, LYS-73, LYS-74, PHE-75, GLN-76, ASP-90, ALA-109, LEU-110, ALA-112, LYS-116, GLN-128, THR-129, VAL-130, ASN-132, GLU-141, PHE-143, TYR-144, PRO-145, HIS-149, GLU-151, TRP-157, PRO-159, ASP-168, LYS-171, SER-172, THR-173, SER-174, ALA-175, LYS-176, ASP-177, LEU-178, GLY-179, ASN-180, THR-191, SER-192, GLU-194, ASN-195, LYS-198, ARG-199, TYR-200, ARG-201, ASN-202, HIS-210, ASP-215, LYS-216, GLY-217, LEU-220, HIS-221, ASP-224. As shown in Fig. 2(a-c), by forming two hydrogen bonds with ASP-516 and LYS-606 of target site.

The RMSF value of these amino acids was lower than 1.5 Å. Additionally, the Propolis Neoflavaniode 1 -PBP-1 complex also participates in hydrophobic interactions, mainly water-mediated bridges, with GLU-141, ASN-180, ASN-195, ARG-199, and GLN-20. Propolis Neoflavaniode 1 interacts with ASP-516 and LYS-606 through hydrogen bonding and water-mediated interactions, as shown in the crystal structure of the protein depicted in Figure. Other interactions were observed with ASP-519, LYS-606, MET-605, THR-610, GLU-523, and LEU-513, but they were not sustained throughout the simulation, Fig. 2(d).

**Fig. 3:**
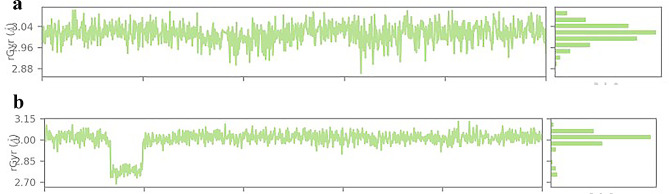
(**a-b**) Gyration radii of the complex. The graph depicts the gyration radii of the complexes formed by Propolis Neoflavaniode-1 with Bmr and Propolis Neoflavaniode-1 with PBP-1 (green) that were determined from molecular dynamics simulations

The RGyr parameter, which was calculated using the entire MD simulation trajectory set, was used to evaluate the compactness of the protein-ligand complexes. When the RGyr value remains unchanged during the simulation, the protein was not altered. The folding variations or structural compactness of Propolis Neoflavaniode-1 with membrane protein Bmr and Propolis Neoflavaniode 1 with PBP-1 complexes were represented by the RGyr parameter during the 100-ns simulation. The average RGyr values for the Propolis Neoflavaniode 1 -Bmr and Propolis Neoflavaniode 1–PBP-1 complexes were 3.1 Å and 2.75 Å, respectively. Notably, significant fluctuations were observed in the RGyr parameter for the PBP complex, whereas no significant fluctuations were observed for the Bmr complex. These findings suggest that both Bmr and PBP remain stable after binding with Propolis Neoflavaniode 1 see Fig. 3(a-b).

### Dynamic cross correlation matrix and PCA analysis

**Fig. 4:**
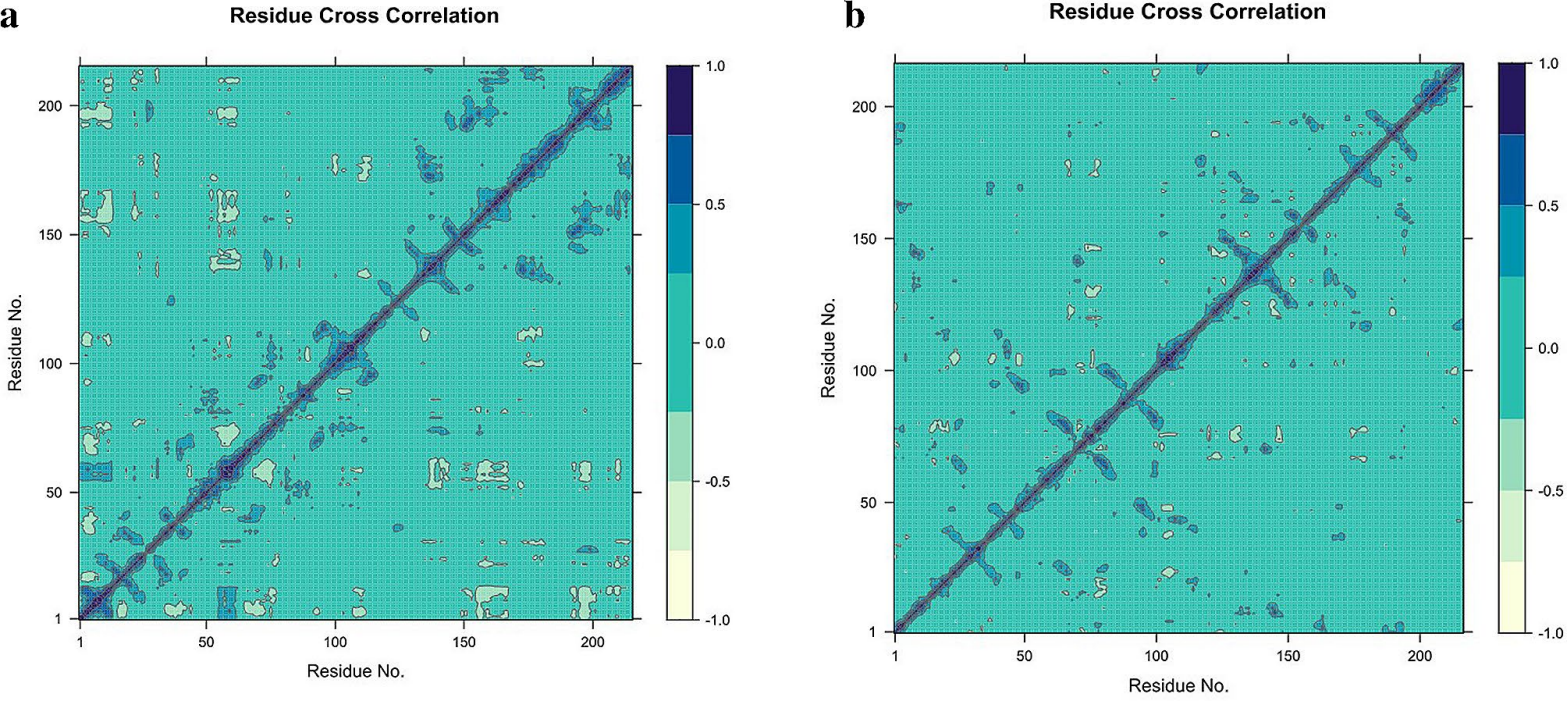
Dynamic cross correlation matrix (DCCM) (**a-b**). (**a**) DCCM plot of Propolis Neoflavaniode-1 membrane protein Bmr (*Bacillus subtilis*). (**b**) DCCM plot of Propolis Neoflavaniode-1 -PBP (*Staphylococcus aureus)*

**Fig. 5:**
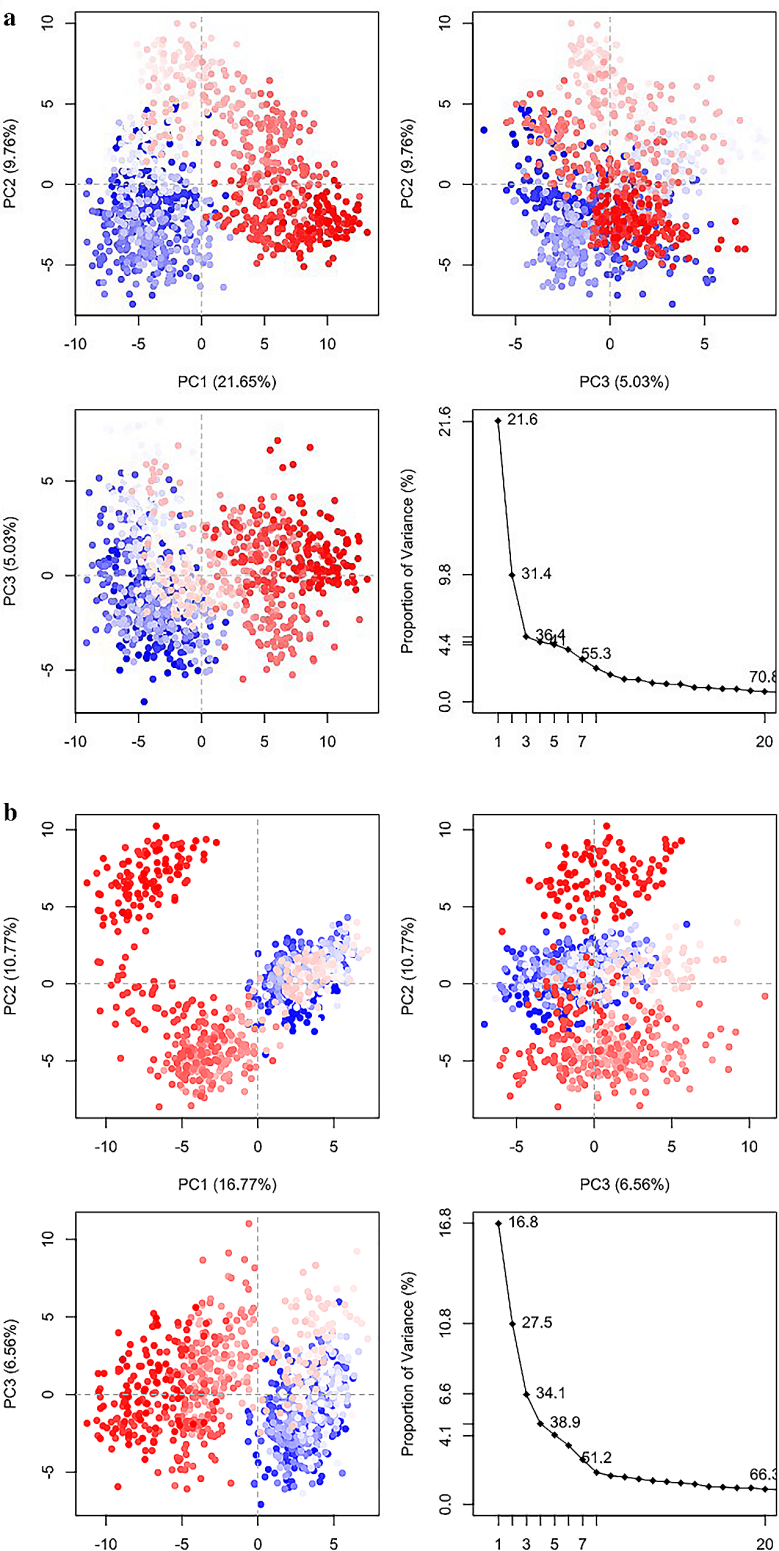
PCA loading Plot (**a-b**). (**a**) PCA plot of Propolis Neoflavaniode-1 with Bmr complex (*Bacillus subtilis*). (**b**) Propolis Neoflavaniode-1–PBP-1 complex (*Staphylococcus aureus)*: the movement of Apo form (blue) and ligand bound (red) using projections of MD trajectories. The first 20 eigen vectors were plotted versus eigen value for Apo (black) and ligand bound molecule (red)

The analysis of molecular dynamics simulations was conducted to evaluate the dynamic cross-correlation among the domains within protein chains bound with Bmr and PBP-1 with Propolis Neoflavaniode 1. The resulting data was visualized, where the blue blocks indicated the residues with highly correlated movement, and the white blocks indicated the residues with the least correlation. The concerted movement of residues in the Bmr with Propolis Neoflavaniode 1 complex was observed to be primarily confined to the α-helices 20–50, as depicted in the figure. In contrast, the PBP-1 with Propolis Neoflavaniode 1 complex exhibited highly correlated movement among the residues within the 18–45 regions, as depicted in Figs. 4 and 5(a-b).

### In vitro antibacterial analysis

#### MIC of Propolis Neoflavaniode 1 samples group 1–4 (gram positive bacteria)

MIC, the minimum inhibitory concentration was calculated for Propolis Neoflavaniode-1 against all bacterial strains. According to our results show that in group-I, *Bacillus subtilis were shown significantly lower MIC values of* Propolis Neoflavaniode 1; P1 (MIC 0.02 mg/mL), P4 (MIC 0.02 mg/mL), P9 (MIC 0.03 mg/mL). *In group-II*,* III*,* and VI*,* were shown significantly lower MIC values of* Propolis Neoflavaniode 1 in P14 (MIC 0.02 mg/mL), P23 (MIC 0.03 mg/mL), P31 (MIC 0.02 mg/mL), and P35 (MIC 0.03 mg/mL).

In group-I, *Staphylococcus aureus* pathogen were significantly reduced and showed MIC value in P2 (MIC 0.02 mg/mL), P4 (MIC 0.0.02 mg/mL), P9 (MIC 0.03 mg/mL). In group-II, III, and VI, P11 (MIC 0.02 mg/mL), P13( MIC 0.03 mg/mL), P20 (MIC 0.02 mg/mL), P28 (MIC 0.03 mg/mL), P31 (MIC 0.03 mg/mL), P32 (MIC 0.03 mg/mL), P33 (MIC 0.02 mg/mL), P34 (MIC 0.04 mg/mL), and P35 (MIC 0.02 mg/mL). Lower MIC values means more active was the sample against the pathogen. Propolis-neoflavaniode-1 were shown highly significant results against gram positive bacteria (*Bacillus subtilis and Staphylococcus aureus)* exhibited susceptibility to samples *p* < 0.001.

#### MIC of Propolis Neoflavaniode 1 samples group 1–4 (gram negative bacteria)

According to our results show that in group-I- II, III, and VI, *Pseudomonas aeruginosa* were shown less significantly lower MIC values of propolis-Neoflavaniode-1, P3 (MIC 0.06 mg/mL), P5 (MIC 0.07 mg/mL), P7 (MIC 0.07 mg/mL), P14 (MIC 0.07 mg/mL), P23 (MIC 0.06 mg/mL), P31 (MIC 0.06 mg/mL), and P35 (MIC 0.06 mg/mL).

In group-I- II, III, and VI, were shown less *Escherichia coli* significantly lower MIC values of propolis-Neoflavaniode-1 P3 (MIC 0.07 mg/mL), P5 (MIC 0.07 mg/mL), P7 (MIC 0.07 mg/mL), P14 (MIC 0.07 mg/mL), P23 (MIC 0.08 mg/mL), P31 (MIC 0.07 mg/mL), and P35 (MIC 0.06 mg/mL).

**Fig. 6:**
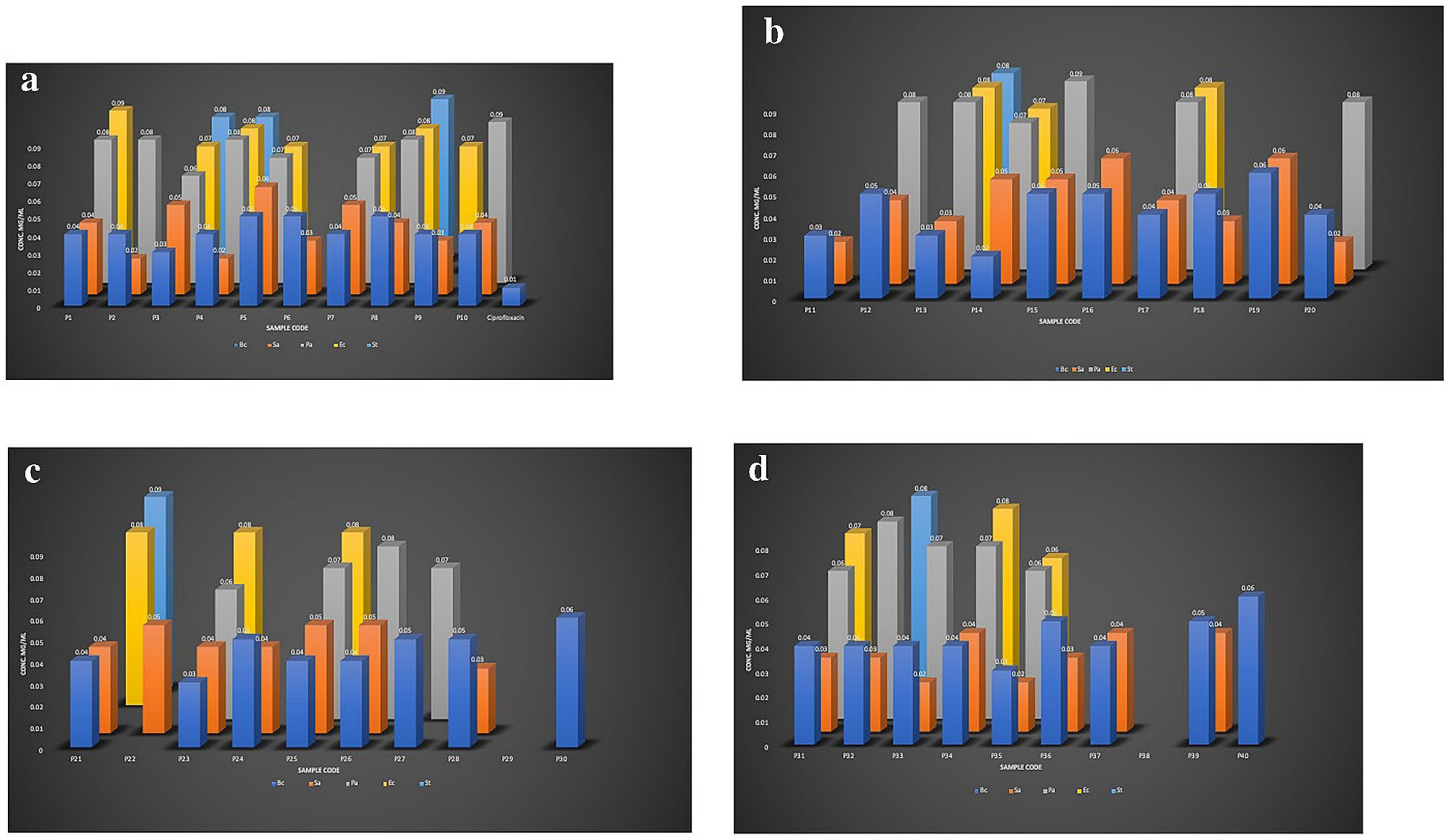
(**a-d**) Bar graph represent Minimum inhibitory Concentration (MIC). (**a**) MIC of Propolis Neoflavaniode-1 samples in group 1 (North Punjab). (**b**) MIC of Propolis Neoflavaniode-1 samples in group 2 (South Punjab). (**c**) MIC of Propolis Neoflavaniode-1 samples in group 3 (Central Punjab). (**d**) MIC of Propolis Neoflavaniode-1 samples in group 4 (West Punjab)

In group-I- II, III, and VI, Propolis Neoflavaniode-1 were inactive against *Salmonella typhimurium*, therefore its MIC was not calculated, see in Fig. 6(a-d). Propolis Neoflavaniode-1 were shown less significant results against gram negative bacteria (*Pseudomonas aeruginosa*,* Escherichia coli* and *Salmonella typhimurium*).

#### Propolis Neoflavonoide-1 combination with ciprofloxacin against bacterial infection

**Fig. 7:**
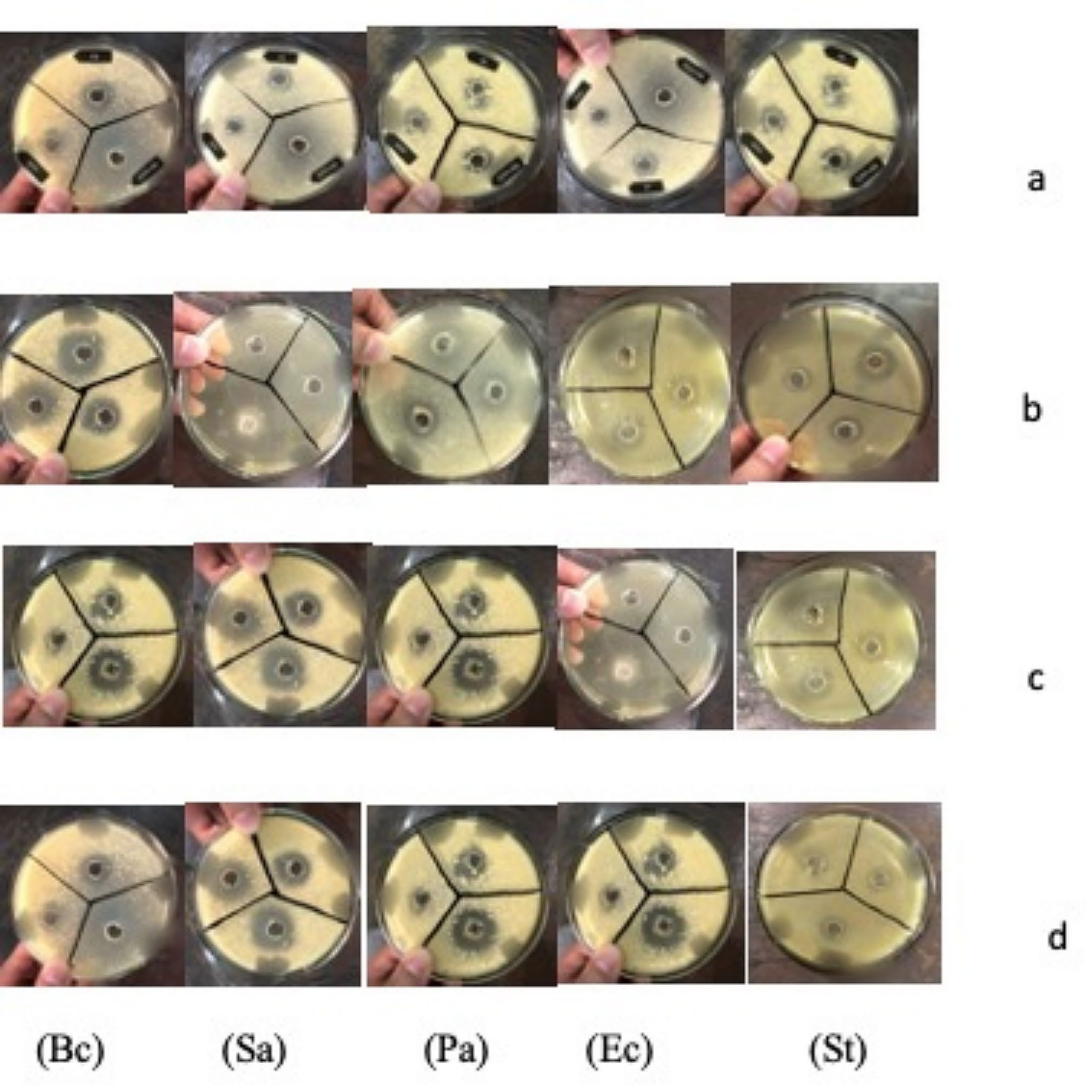
**(a-d)**: (**a**) Zone of inhibition (mm) of P 3 + Ciprofloxacin against 5 bacterial strains. (**b**) Zone of inhibition (mm) of P 13 + Ciprofloxacin against 5 bacterial strains. (**c**) Zone of inhibition (mm) of P 28 + Ciprofloxacin against 5 bacterial strains. (**d**) Zone of inhibition (mm) of P 35 + Ciprofloxacin against 5 bacterial strains

The P3, P13, P28, and P35 samples were experimentally evaluated against different bacterial strains using Propolis Neoflavonoide 1 and Ciprofloxacin (1:1); the results showed a significant increase in the zone of inhibition, specifically against Gram-positive bacteria (*p* < 0.005). However, very little improvement was seen against Gram-negative bacteria. The sample was more active against the pathogen when the MIC value was lower. All of the gram-negative bacteria samples had little to no action at lower concentrations against *Escherichia coli* (MIC < 0.5) and *Pseudomonas* (MIC < 0.5). It was found that *Salmonella typhimurium* had the highest degree of resistance, see Fig. 7(a-d).

Upon applying Shapiro-Wilk test, the data appeared to be non-normal as shown in below Table 3 (*p* < 0.05).

**Table 3:** Shapiro-Wilk test on bacteria

Bacteria	Statistic	Sig.
*Bacillus subtilis*	0.813	0.000
*Staphylococcus aureus*	0.914	0.005
*Pseudomonas aeruginosa*	0.735	0.000
*Escherichia coli*	0.672	0.000
*Salmonella typhimurium*	0.438	0.000

As data was non-normal so applying Kruskal-Wallis test for group comparisons also showed insignificant results on comparing the bacteria MIC values across the four regions (Table 4).

**Table 4:** Kruskal-Wallis test for group comparison of the bacteria MIC values across the four regions of Punjab

Bacteria	Punjab Region	Mean ± Standard Deviation	Kruskal Walis Test	*p*-Value
*Bacillus subtilis*	Central	0.036 ± 0.021	0.224	0.974
	North	0.042 ± 0.006		
	South	0.042 ± 0.015		
	West	0.039 ± 0.02		
*Staphylococcus aureus*	Central	0.03 ± 0.022	4.43	0.218
	North	0.038 ± 0.013		
	South	0.04 ± 0.015		
	West	0.025 ± 0.015		
*Pseudomonas aeruginosa*	Central	0.028 ± 0.036	6.26	0.100
	North	0.061 ± 0.03		
	South	0.048 ± 0.041		
	West	0.034 ± 0.036		
*Escherichia coli*	Central	0.024 ± 0.039	4.32	0.229
	North	0.053 ± 0.037		
	South	0.023 ± 0.037		
	West	0.021 ± 0.034		
*Salmonella typhimurium*	Central	0.009 ± 0.028	2.29	0.514
	North	0.025 ± 0.04		
	South	0.008 ± 0.025		
	West	0.008 ± 0.025		

The above table showed that the mean MIC value for bacteria across the regions, regarding *Bacillus subtilis* in central region samples, the mean MIC was 0.036 ± 0.021, for northern region samples, the mean was 0.042 ± 0.006, for Southern region, the mean was 0.042 ± 0.015 and for Western region, the mean was 0.039 ± 0.02. The mean difference was statistically insignificant (*p* = 0.974). Regarding *Staphylococcus aureus*, in central region samples, the mean MIC was 0.03 ± 0.022, for northern region samples the mean was 0.038 ± 0.013, for Southern region the mean was 0.04 ± 0.015 and for Western region the mean was 0.025 ± 0.015. The mean difference was statistically insignificant (*p* = 0.218). Regarding *Pseudomonas aeruginosa*, in central region samples, the mean MIC was 0.028 ± 0.036, for northern region samples the mean was 0.061 ± 0.03, for Southern region the mean was 0.048 ± 0.041 and for Western region the mean was 0.034 ± 0.036. The mean difference was statistically insignificant (*p* = 0.100). Regarding *Escherichia coli*, in central region samples, the mean MIC was 0.024 ± 0.039, for northern region samples the mean was 0.053 ± 0.037, for Southern region the mean was 0.023 ± 0.037 and for Western region the mean was 0.021 ± 0.034. The mean difference was statistically insignificant (*p* = 0.229). Regarding *Salmonella typhimurium*, in central region samples, the mean MIC was 0.009 ± 0.028, for northern region samples the mean was 0.025 ± 0.04, for Southern and Western region, the mean was 0.008 ± 0.025 each. The mean difference was statistically insignificant (*p* = 0.514).

## Discussion

Propolis Neoflavaniode-1 has been found to possess antimicrobial properties that help protect hives from diseases caused by bacteria, fungi, and yeast. It also acts as a barrier against predators and other external factors. As a result of its antibacterial qualities, research has been conducted on its potential benefits for human health. The ongoing research examines Propolis Neoflavaniode-1 bioactive components and their potential applications, such as antibacterial, antifungal, and antioxidant properties. Hence, Propolis Neoflavaniode 1 made by *Apis mellifera* contains polyphenolic chemicals. The primary polyphenols in propolis, flavonoids, were influenced by the source and the ecological and botanical surroundings in which the bee resides (Almuhayawi 2020; Shehata et al. 2020).

It has been found that propolis Neoflavaniode 1 works well against a variety of bacteria, including those that were both Gram-positive and Gram-negative. Research have shown that it is effective against *Salmonella typhimurium*,* Pseudomonas aeruginosa*,* Bacillus subtilis*,* Staphylococcus aureus*,* and Escherichia coli* (Bouchelaghem 2022). However, it takes time to screen compounds in vitro for toxicity and antibacterial efficacy. Therefore, *in silico* computational methodologies over the past ten years, technologies connected to cheminformatics, molecular docking, and artificial intelligence have grown in popularity in the field of drug design, development, and discovery. There were already a number of ligand- and structure-based molecular docking methods available to facilitate high-throughput drug discovery.

In this study, five compounds (Propolis Neoflavaniode-1, Carvacrol, Cinamaldehyde, Thymol, p-benzoquinone) were molecularly docked to the gram positive and gram negative bacterial outer membrane molecule (Bmr, PBP-1, Dehydratase, ompC, Dispersin of *Staphylococcus aureus*,* Bacillus subtilis*,* Pseudomonas aeruginosa*,* Escherichia coli*,* Salmonella typhimurium strains*, respectively (Bahr et al. 2021). In vitro antibacterial efficacy of these compounds against MDR-strains was further confirmed by data obtained by docking techniques (Abishad et al. 2021).

Based on our *in silico* research, the potential antibacterial properties of specific compounds were assessed through docking analysis, it illustrated the chemicals’ binding energy to the bacterial protein molecule. This analysis identified key residues involved in hydrogen bonding, atomic contact energy, and ligand transformation after refinement (George et al. 2019). Our findings indicate that Propolis Neoflavonoide-1, Carvacrol, Cinamaldol, Thymol, p-benzoquinone, and the standard drug (S*) exhibit strong binding affinities to specific bacterial outer membrane proteins, suggesting that these compounds may be able to inhibit bacterial activity. Propolis Neoflavonoide-1, in particular, interacts with the binding pocket of Bmr, PBP-1, Dehydratase, ompC, and Dispersin to form hydrogen bonding, indicating a strong potential for inhibitory interactions that could prevent the activity of the binding site. These results suggest that Propolis Neoflavonoide-1 has a strong potential to improve the antimicrobial activity of both gram-positive and gram-negative bacteria. Our research indicates that Propolis Neoflavonoide-1 displays superior binding performance compared to other compounds, such as Thymol, Carvacrol, Cinamaldehyde, and p-benzoquinone, when tested against beta-lactamase II and PBP-1. Therefore, these in silico computational data should be validated through in vitro antimicrobial efficacy studies (George et al. 2019; Fatima et al. 2023).

Pharmacokinetic characteristics and toxicity were commonly encountered obstacles when developing medication candidates for clinical trials. The pharmacokinetics of other drugs may be impacted by interactions between drug candidates. Our target compounds, including Propolis Neoflavonoide-1, Carvacrol, Cinamaldehyde, Thymol, and p-benzoquinone, all had good lipophilicity ranges (LogP < 5), with the exception of ciprofloxacin. Regarding TPSA values, it was often observed that natural compounds with lower TPSA values (< 140 Å²) have greater oral bioavailability due to their drug-likeness and bioavailability. All the target compounds were within this range (Adamovich et al. 2020). Certainly, the initial phases of drug development involve identifying appropriate drug candidates with desired pharmacokinetic and drug-like qualities. However, few studies have examined their ADME profiles in relation to in vitro antimicrobial efficacy against MDR bacteria. In the current study, several ADME characteristics, including physicochemical properties, pharmacokinetics, and follow Lipinski’s rule five, were analyzed using the Swiss ADME server for the five compounds with S* drug identified *in silico* ADME prediction. These compounds demonstrated drug-likeness, as evidenced by their bioavailability score of 0.55 and absence of any violations of Lipinski’s rule of five in Table 1 (Hassan et al. 2022).

Enzymes P-gp and CYP-450 help the body to break down and remove foreign substances, which lowers their potential toxicity and protects tissues. Together, these systems prevent toxic substances from building up in tissues, acting as a buffer against the negative effects of numerous foreign toxins or drugs. In this study, Propolis Neoflavonoide-1 and other natural compounds had no P-gp inhibitors (Doğanay et al. 2022). Protox-II, StopTox assesses a given molecule’s possible toxicity using machine learning algorithms and to assess a compound’s chemical structure and its toxicity, with a particular emphasis on hepatotoxicity (liver toxicity). None of the compounds showed liver or immunotoxicity (Banerjee et al. 2018; Ghosh et al. 2019). *In silico* acute toxicity analysis of natural compounds tested with the reference drug Ciprofloxacin by Stop Tox showed dermal and oral toxicity, except Propolis Neoflavonoide-1 and S*, with a nontoxic range of confidence score (≤ 50%). While *in silico* toxicity predictions can provide significant insights and assist in the selection of compounds in the early phases of drug discovery, it was crucial to validate these predictions using in vitro safety assays before advancing to in vivo research utilizing laboratory animal models in Table 2 (Ferreira and Andricopulo 2019).

Because of its remarkable free-energy interactions with the gram-positive bacterial protein motif, which includes *Staphylococcus aureus and Bacillus subtilis*, Propolis neoflavanoide-1 was chosen. RMSD of protein C-α atoms, RMSD of ligand atoms, RMSF, and protein-ligand contact histogram were among the metrics that were calculated using the MD simulation trajectory. The simulation revealed that the Propolis Neoflavonoide-1 -protein complex remained stable, as evidenced by the RMSD of Bmr of the C-α backbone under 3 Å than PBP-1. The ligand complex’s RMSD with Bmr minor fluctuated significantly at first but stabilized after roughly 60 ns of simulation. Throughout the remainder of the simulation, the complexes stayed stable. The covariance matrix’s eigenvalues, which the PCA approach used to determine the total combined motion of the C-atoms in the protein ligand complexes. The time-dependent switching between different conformations during the simulation has shown by the continuous red-to-blue color transition, where dots that begin and end with red represent the alignment of each frame. These findings show the best and highest % age of effective PCA analysis of gram positive bacteria was at clear complex conformational dynamics, which can help with the creation of new antimicrobial drugs. (Ferreira and Andricopulo 2019; Tanreh et al. 2023).

Propolis Neoflavonoide-1 has been demonstrated to display bactericidal properties. This substance can harm the cytoplasm or cell wall of bacteria, inhibiting protein synthesis and preventing their division. Additionally, it enhances an organism’s natural defenses and immunological system. The agar well diffusion technique was used to evaluate the antimicrobial activity of each Propolis Neoflavonoide 1 sample against three gram-negative bacteria *(Pseudomonas aeruginosa*,* Escherichia coli*,* and Salmonella typhimurium)* and two gram-positive bacteria *(Staphylococcus aureus and Bacillus subtilis).* These bacteria were chosen based on their ability to cause common diseases within the local population, including foodborne illnesses, diarrhea, skin infections, urinary tract infections, respiratory system infections, vomiting, and fever. In our study, the antibacterial efficacy of group 1–4 was assessed using MIC values for different groups (Zulhendri et al. 2021; Bhatti et al. 2023; Din et al. 2023). The MIC values of the Propolis Neoflavonoide-1 compound examined for gram-positive strains ranged from 0.02 to 0.05 mg/ml, while those for gram-negative strains were 0.06 to 0.09 mg/ml. The MIC values of the propolis Neoflavonoide-1 under test may be influenced by strain diversity, variations in the virulence factors of the bacteria, or structural variations in the bacterial membranes (Khan et al. 2019). A significant increase in the zone of inhibition, particularly against gram-positive bacteria, was observed during the experimental evaluation of the P3, P13, P28, and P35 samples in combination with ciprofloxacin against a variety of bacterial species. However, little progress was made in gram-negative bacteria (Liu et al. 2020).

The results indicated that most samples had limited impact on gram-negative bacteria, whereas gram-positive bacteria were significantly inhibited, as shown by the presence of a substantial zone of inhibition (measured in millimeters). The observed antibacterial activity of the samples exhibited a positive correlation with concentration, that was, the antimicrobial effect increased with increasing concentration. The efficacy of Propolis Neoflavonoide-1 was compared with that of ciprofloxacin against a range of bacterial isolates. Ciprofloxacin, a broad-spectrum fluoroquinolone antibiotic, was selected as the standard drug S* owing to its efficacy against both gram-positive and gram-negative bacteria (Suleman 2016; AMIN et al. 2023; Hassan et al. 2023). It may act by inhibiting the activity of crucial bacterial enzymes, namely topoisomerase IV and topoisomerase II (DNA gyrase), which play vital roles in the separation of bacterial DNA (Khan et al. 2018; GOHAR et al. 2023; Ullah et al. 2023a, b).

Our investigation led us to conclude that of the studied bacterial strains, *Bacillus subtilis and Staphylococcus aureus were the most susceptible*,* and Salmonella typhimurium* was the most resistant. Additionally, the efficacy of ciprofloxacin increased with the administration of Propolis Neoflavonoide-1. This synergistic effect may encourage the development of novel antibacterial combinations using natural ingredients. In addition, there was a strong correlation between the in vitro antimicrobial tests and observed *in silico* docking results. We can conclude that the bioactive compound of Propolis Neoflavonoide-1 can be potential therapeutic compound for antibacterial infection. The Propolis Neoflavonoide-1 compound can also be used in combination with antibacterial inhibitor (Ciprofloxacin) to increase potency and efficacy of bacterial treatment. This synergistic effect may encourage the development of novel antibacterial combinations using natural ingredients. The results obtained from the recent studies support the use of natural compound Propolis Neoflavonoide 1 act as an antibacterial medicine, which was strongly support and correlate by in vitro-experiments.

### Electronic supplementary material

Below is the link to the electronic supplementary material.

Supplementary Material 1

Supplementary Material 2

Supplementary Material 3

Supplementary Material 4

Supplementary Material 5

Supplementary Material 6

## Data Availability

No data associated in the manuscript.
