# Supplementary material for: Antibacterial potential of Propolis: molecular docking, simulation and toxicity analysis
Source: AMB Express. 2024 Jul 16;14:81. doi: 10.1186/s13568-024-01741-0 (PMC11252112; doi:10.1186/s13568-024-01741-0)
Supplement: Supplementary file 1 — Supplementary Material 1 [file 13568_2024_1741_MOESM1_ESM.docx]

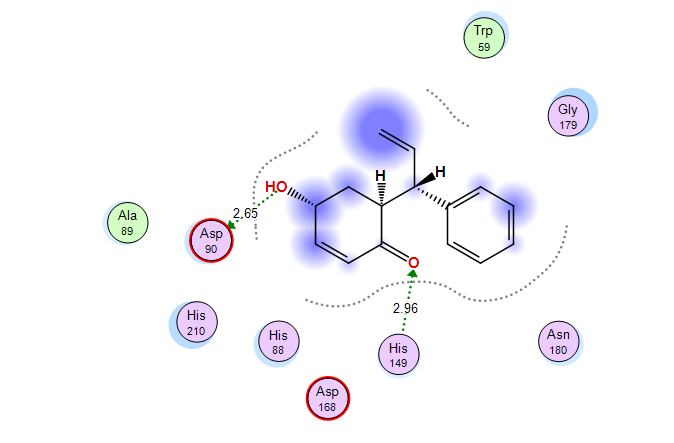

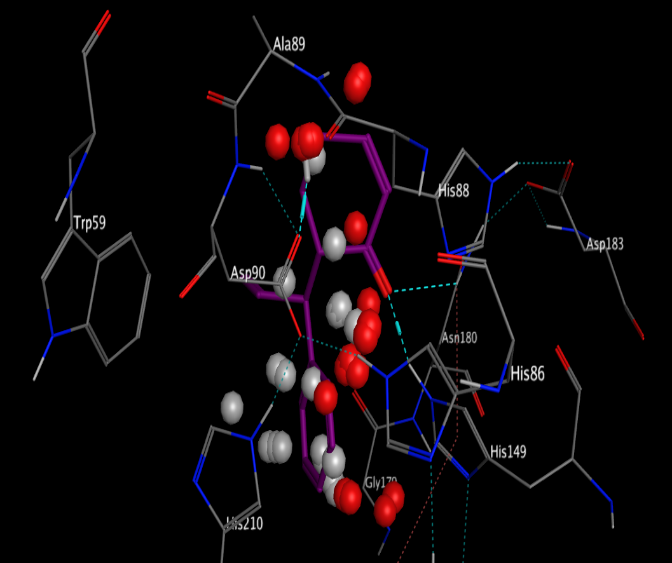


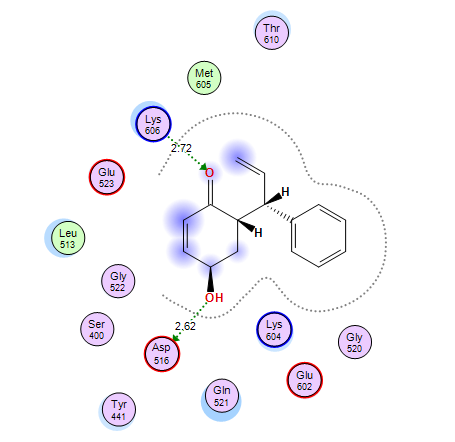

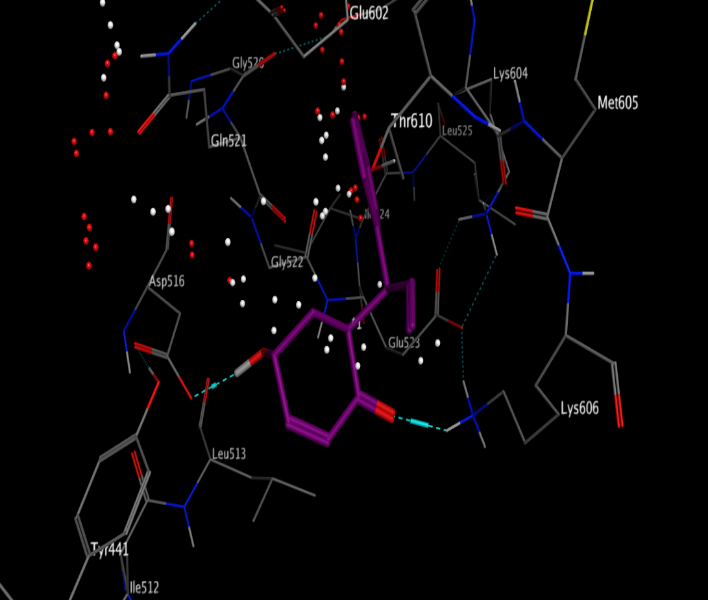


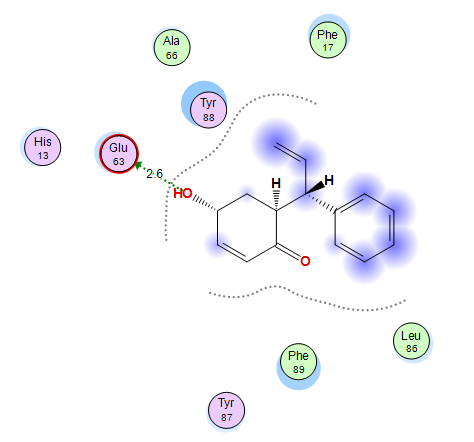

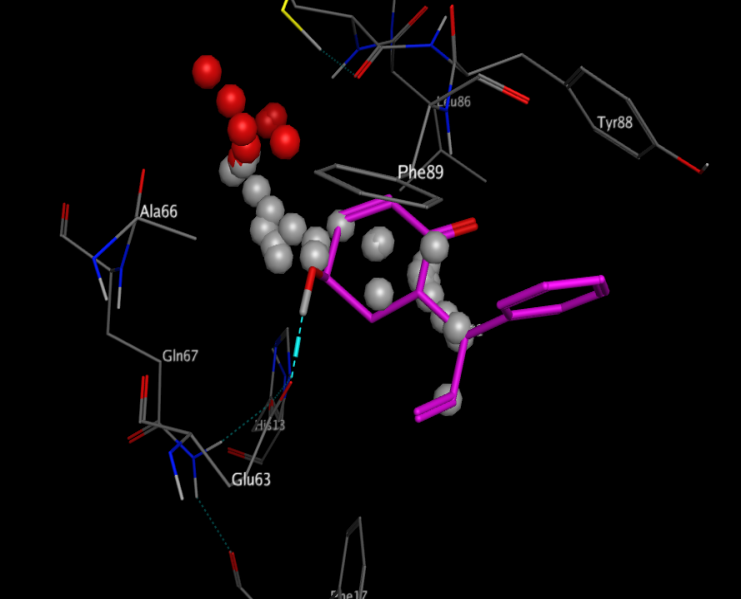


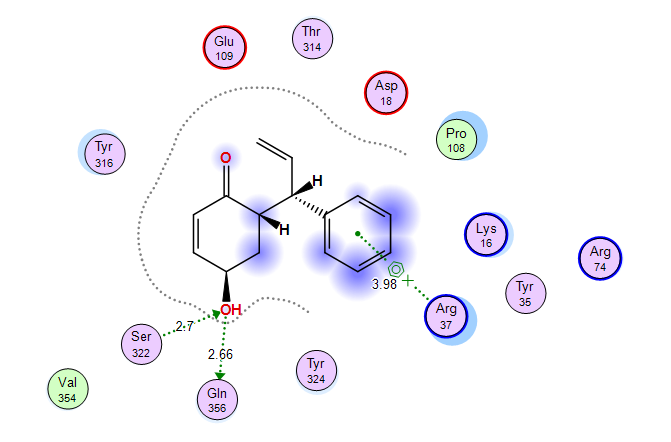

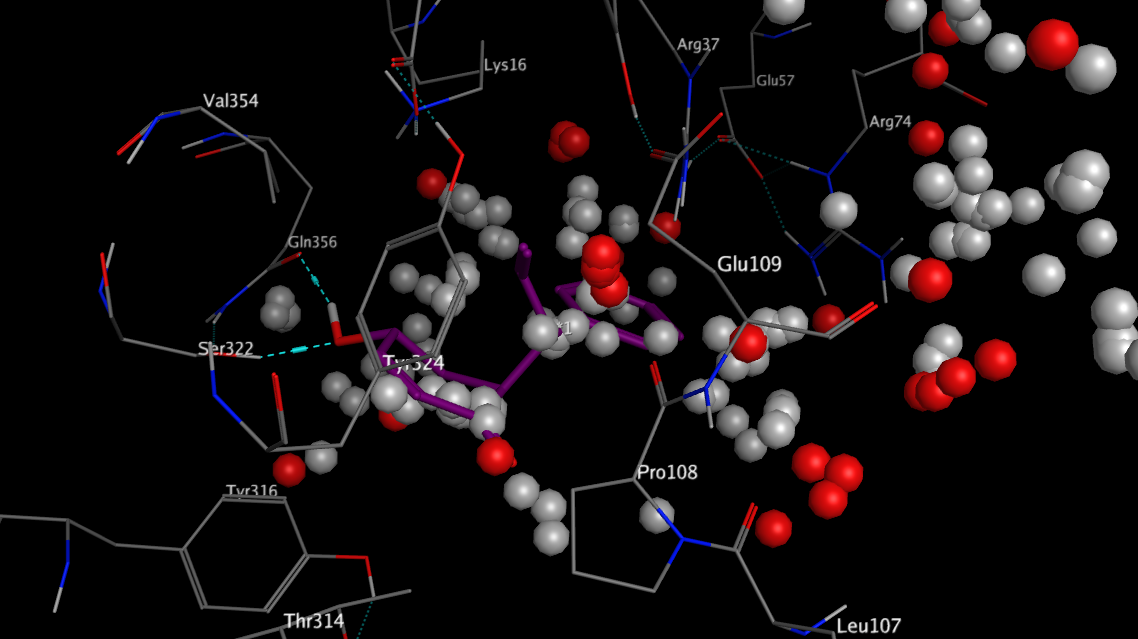


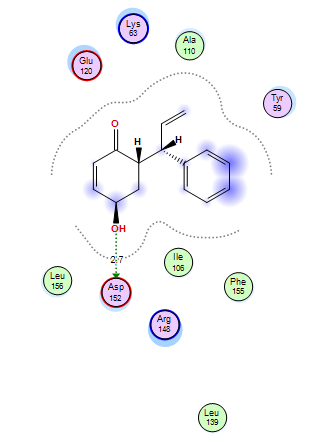

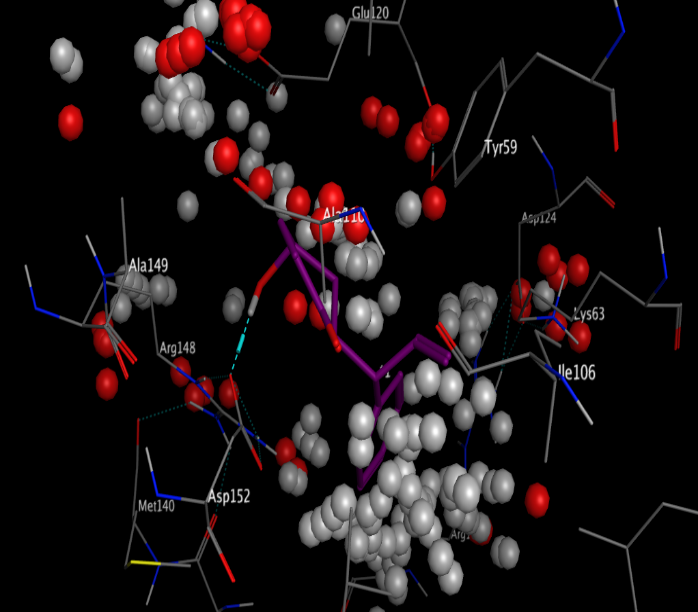


**Supplementary Figure 1 (a-j). Propolis Neoflavaniode-1 –bacterial target molecule complex 2D graph:** Ligand color show purple. (a-b) Propolis Neoflavaniode-1 Bmr complex 2D graph. (c-d) Propolis Neoflavaniode-1 - PBP-1 complex. (e-f) Propolis Neoflavaniode-1 -Dehydratase complex 2D graph. (g-h) Propolis Neoflavaniode-1 – ompC complex 2D graph. (i-j) Propolis Neoflavaniode-1- Dispersin complex 2D graph.
