# Supplementary material for: Antibacterial potential of Propolis: molecular docking, simulation and toxicity analysis
Source: AMB Express. 2024 Jul 16;14:81. doi: 10.1186/s13568-024-01741-0 (PMC11252112; doi:10.1186/s13568-024-01741-0)
Supplement: Supplementary file 2 — Supplementary Material 2 [file 13568_2024_1741_MOESM2_ESM.docx]

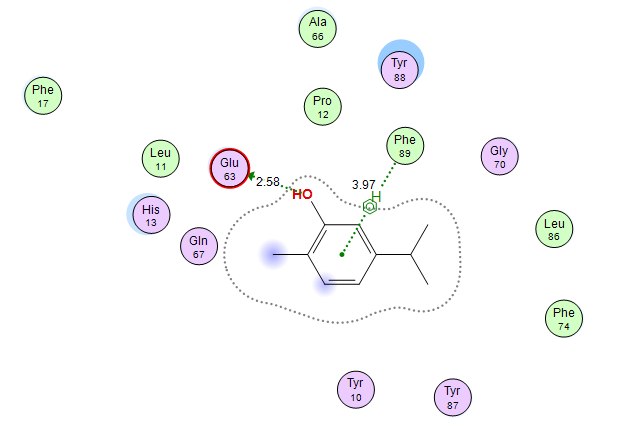

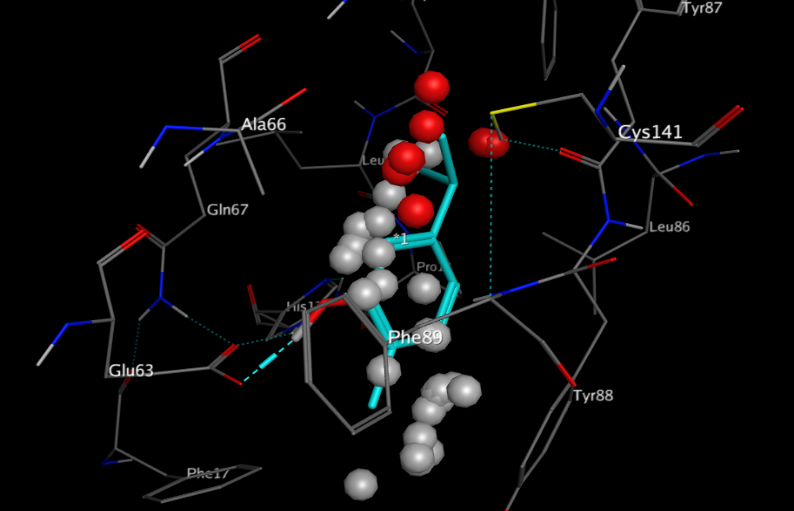


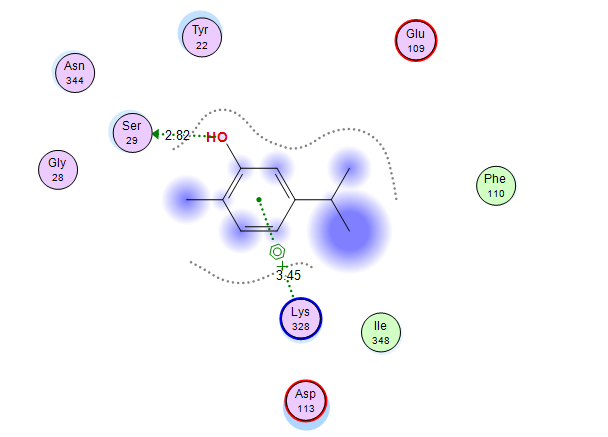

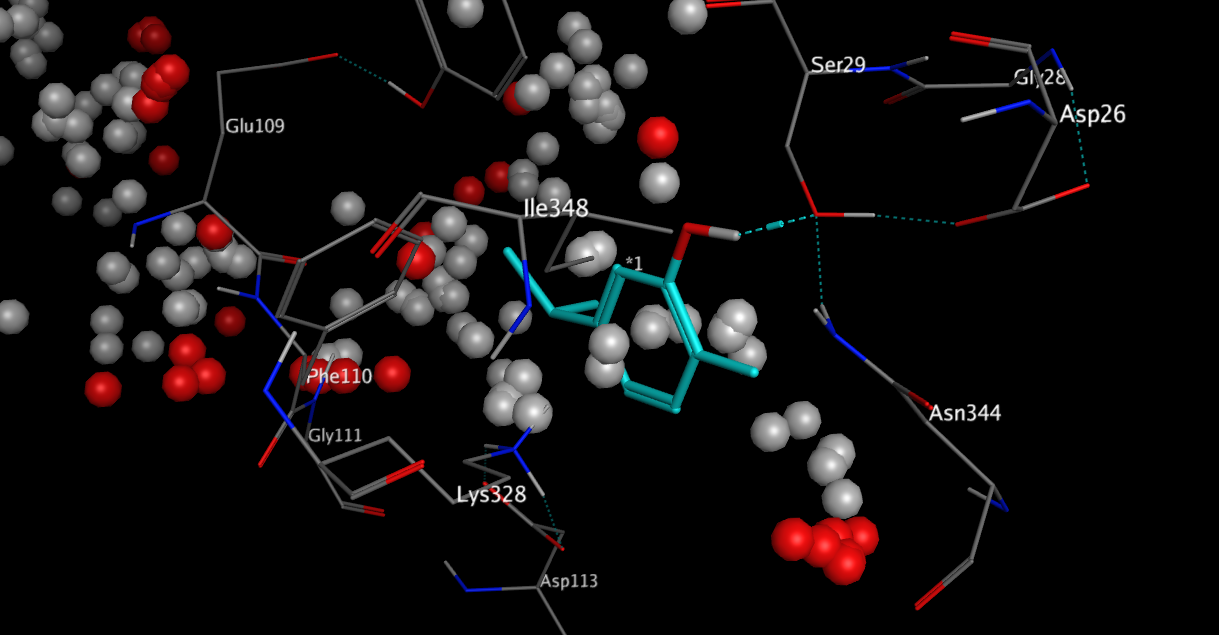


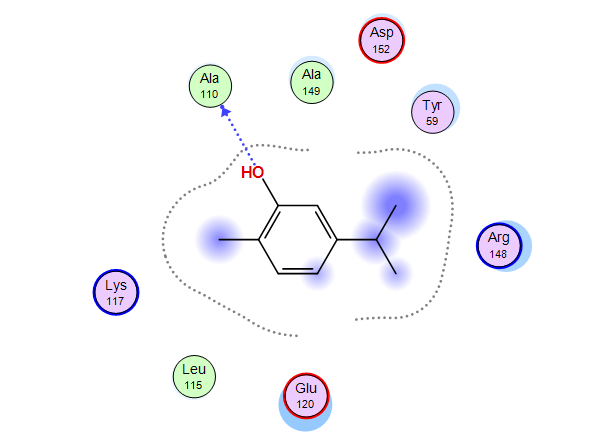

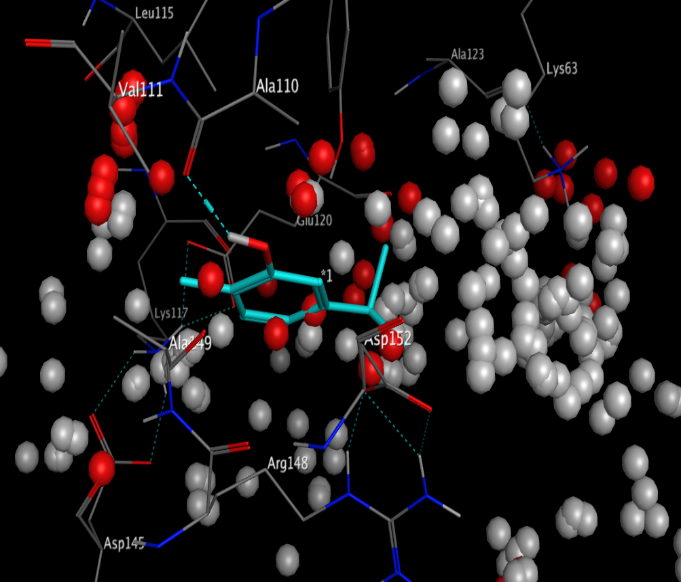


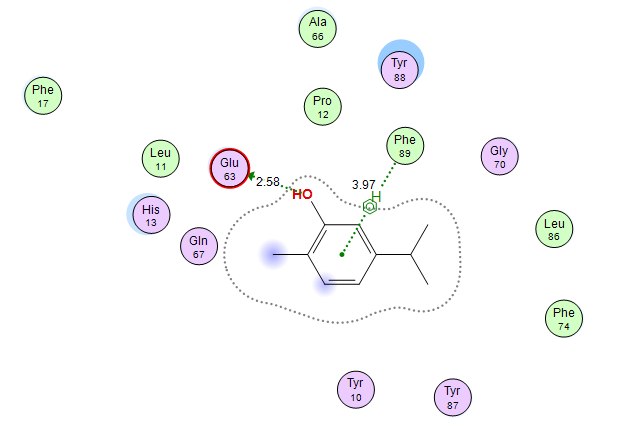

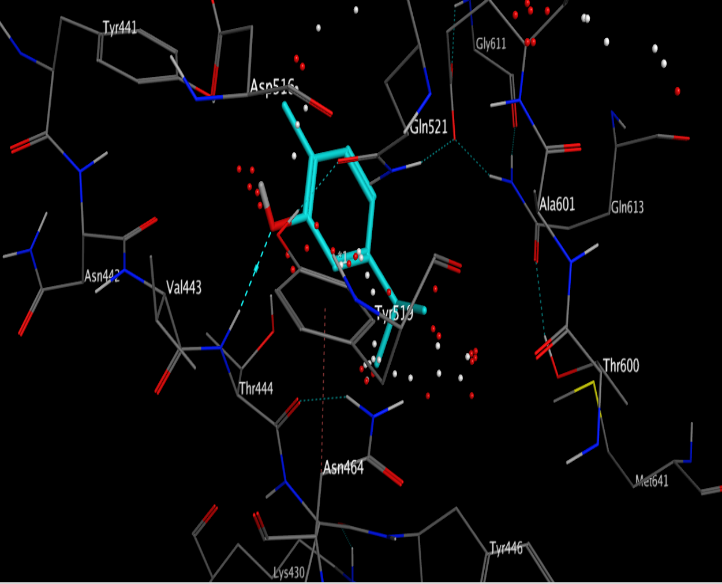


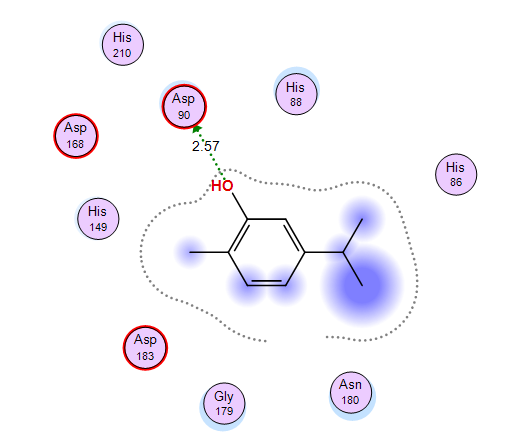

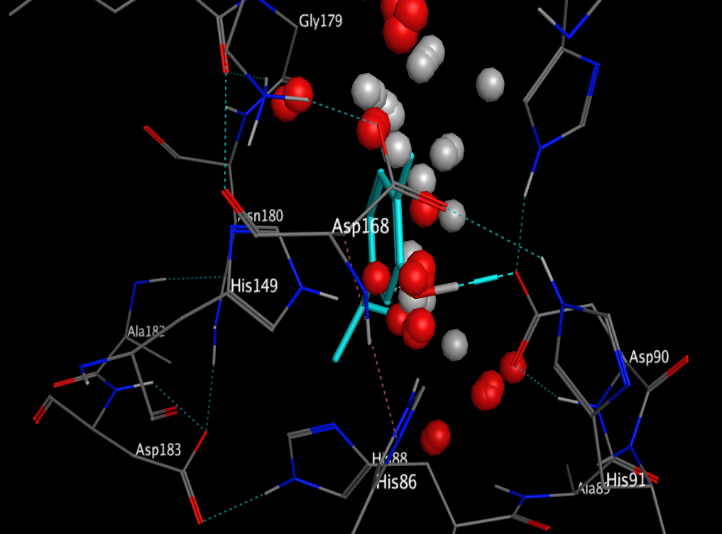


**Supplementary Figure 2 (a-j). Carvacrol–bacterial target molecule complex 2D graph:** Ligand color show cyan (a-b) Thymol- Bmr 2D graph. (c-d) Thymol- PBP-1 complex. (e-f) Thymol-Dehydratase complex 2D graph. (g-h) Thymol- ompC complex 2D graph. (i-j) Thymol- Dispersin complex 2D graph.
