# Supplementary material for: Antibacterial potential of Propolis: molecular docking, simulation and toxicity analysis
Source: AMB Express. 2024 Jul 16;14:81. doi: 10.1186/s13568-024-01741-0 (PMC11252112; doi:10.1186/s13568-024-01741-0)
Supplement: Supplementary file 5 — Supplementary Material 5 [file 13568_2024_1741_MOESM5_ESM.docx]

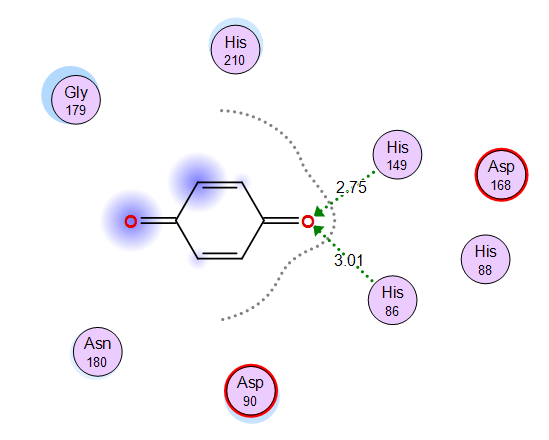

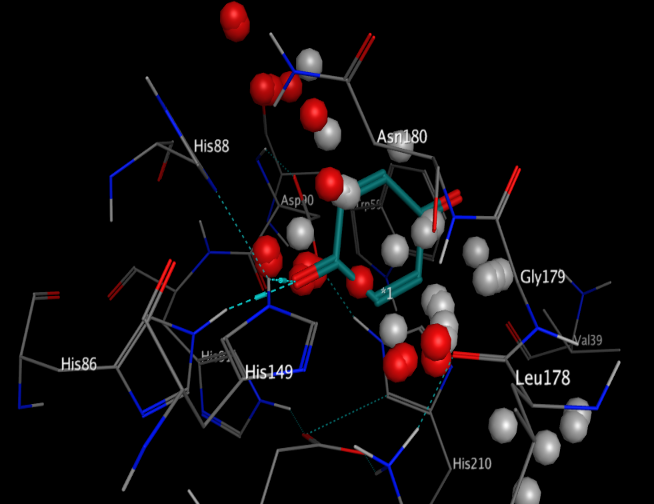


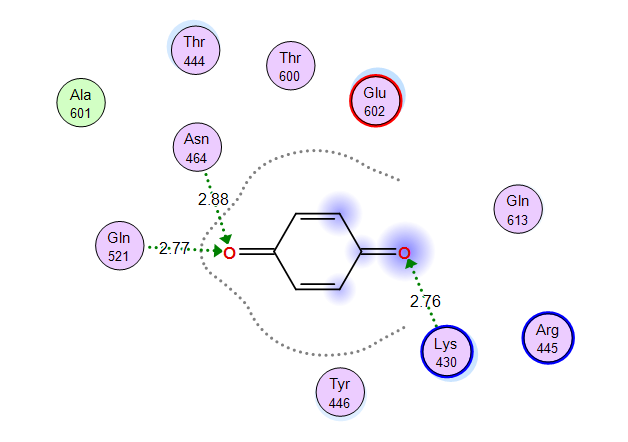

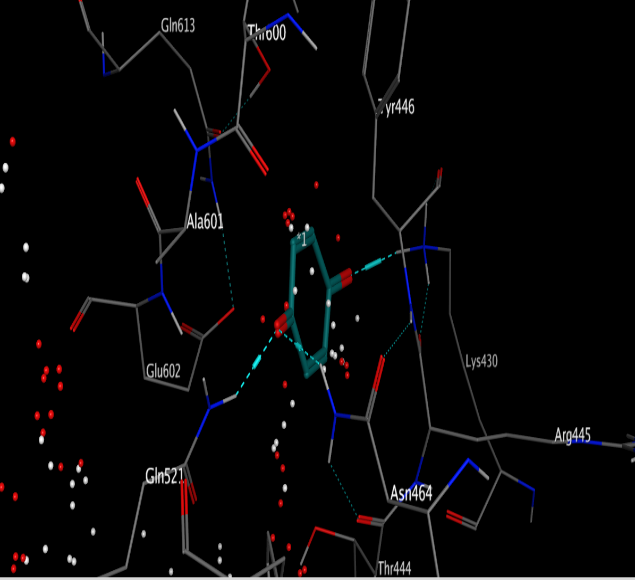


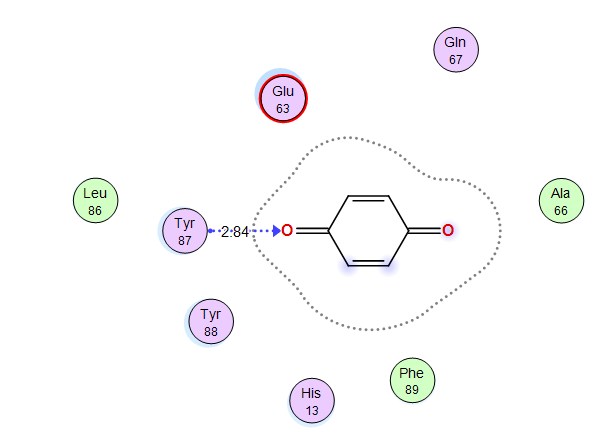

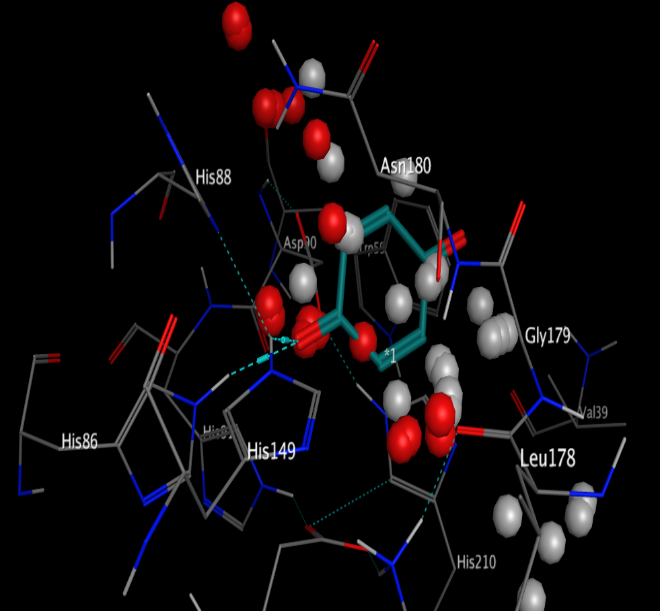


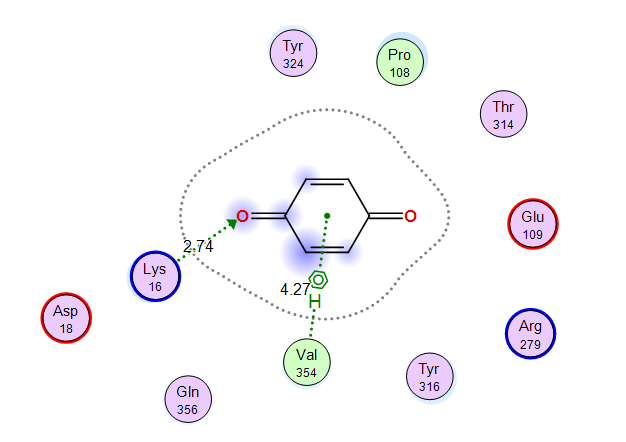

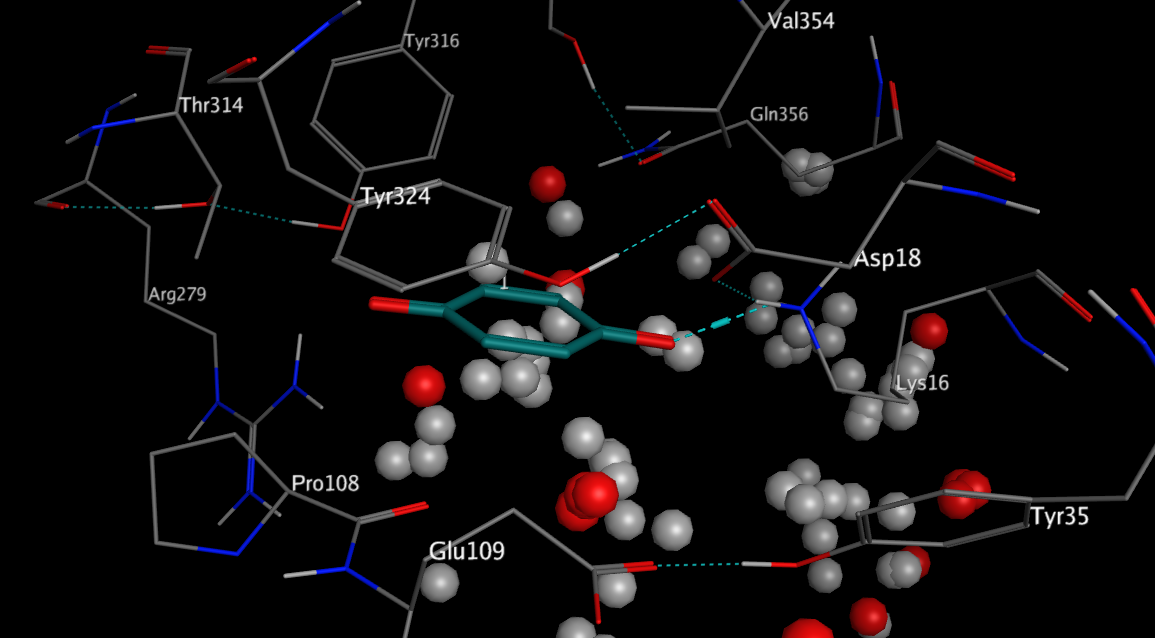


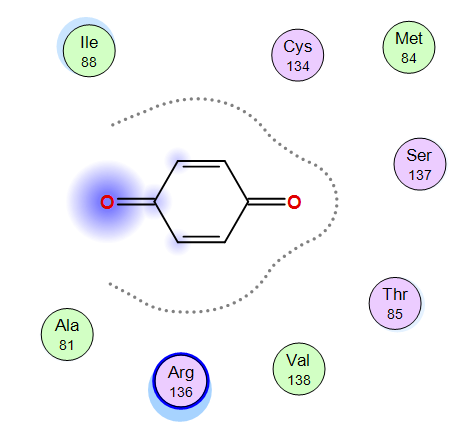

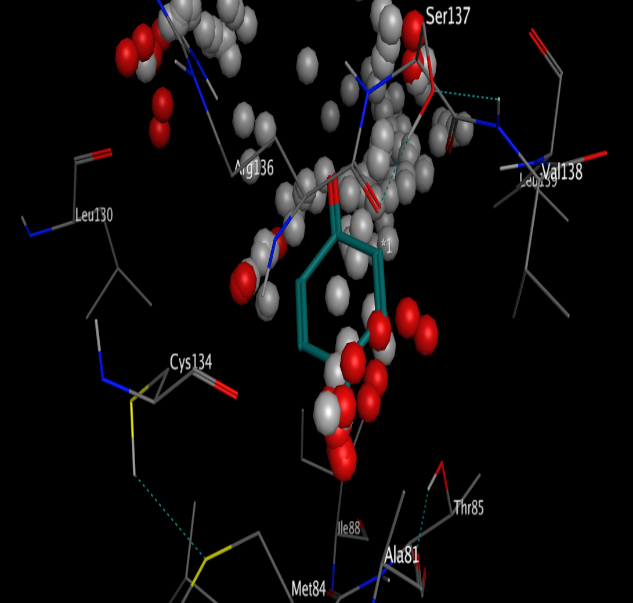


**Supplementary Figure 5 (a-e). p-benzoquinone- bacterial target molecule complex 2D graph:** Ligand color show dark green. (a) p-benzoquinone – Bmr complex 2D graph. (b) p-benzoquinone - PBP complex. (c) p-benzoquinone -Dehydratase complex 2D graph. (d) p-benzoquinone - ompC complex 2D graph. (e) p-benzoquinone- Dispersin complex 2D graph.
