# Supplementary material for: Antibacterial potential of Propolis: molecular docking, simulation and toxicity analysis
Source: AMB Express. 2024 Jul 16;14:81. doi: 10.1186/s13568-024-01741-0 (PMC11252112; doi:10.1186/s13568-024-01741-0)
Supplement: Supplementary file 6 — Supplementary Material 6 [file 13568_2024_1741_MOESM6_ESM.docx]

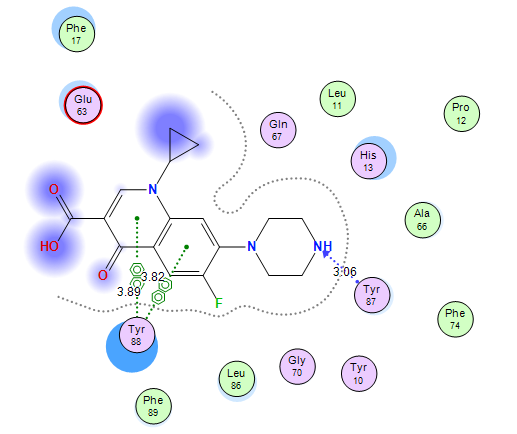

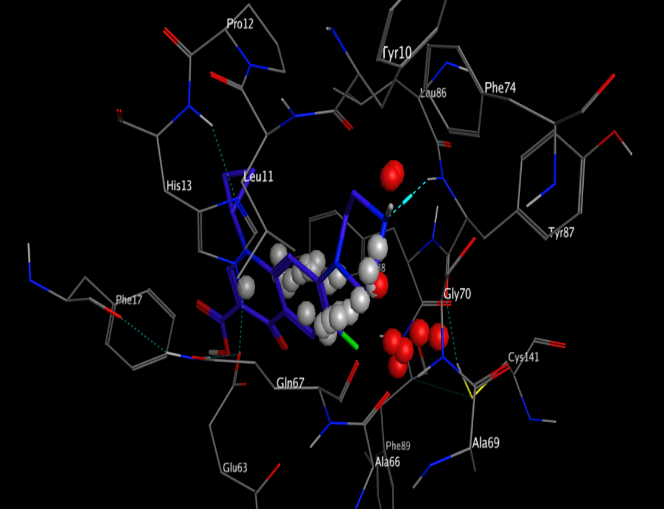


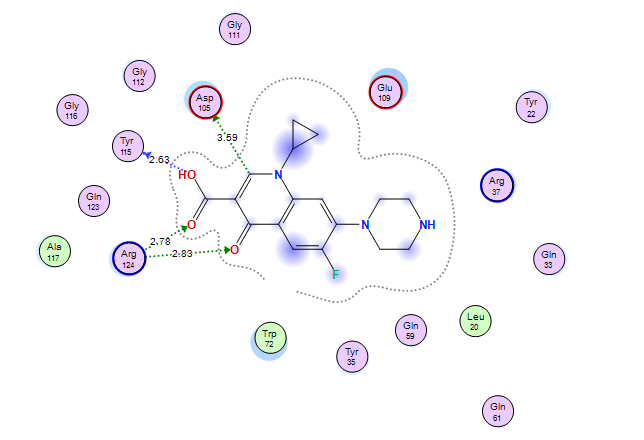

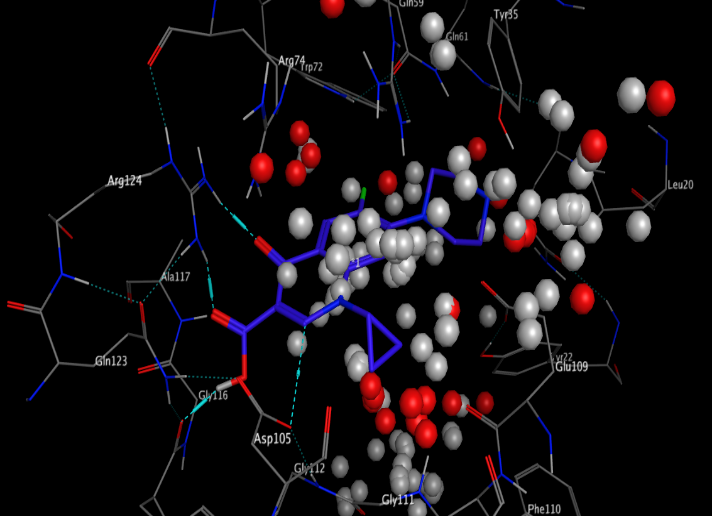


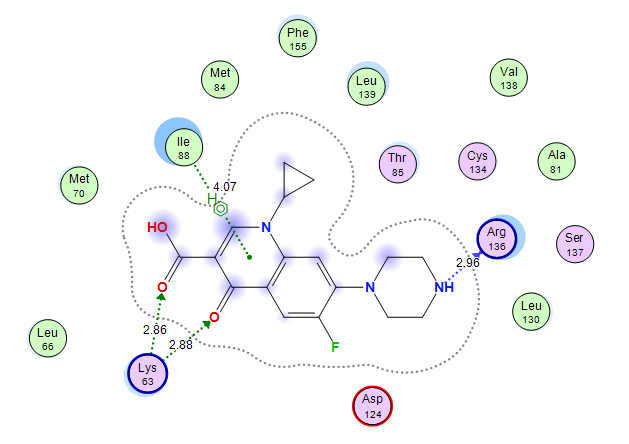

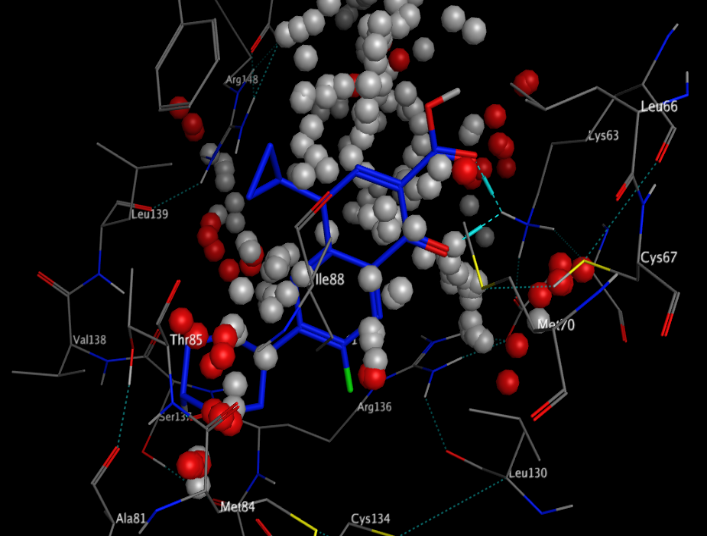


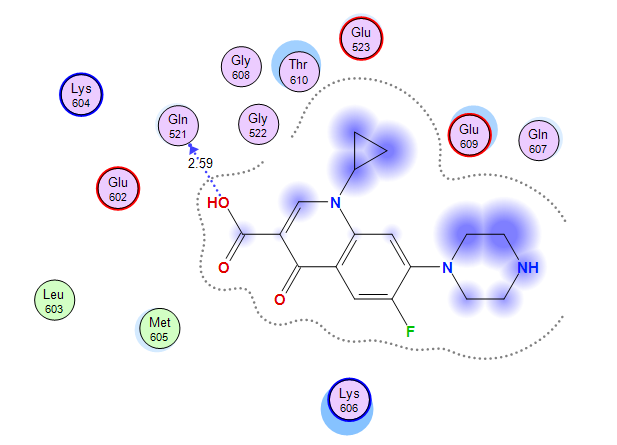

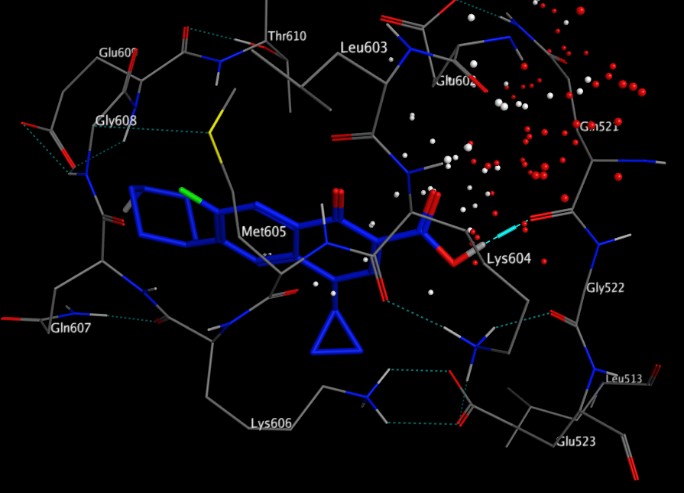


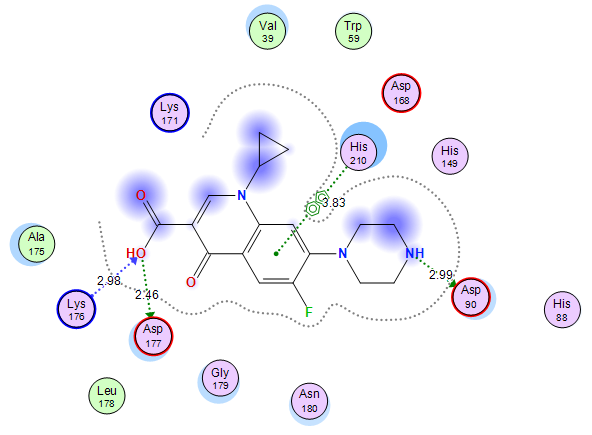

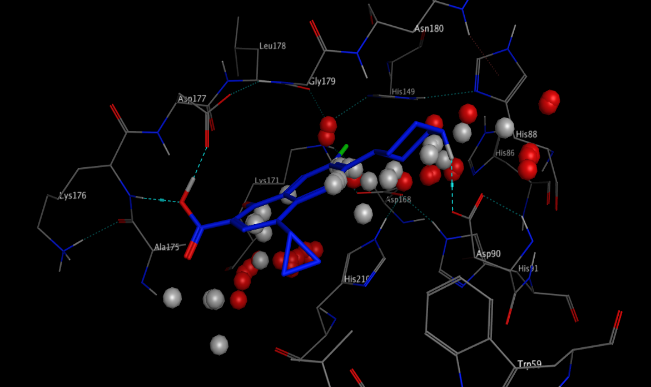


**Supplementary Figure 6 (a-j). Ciprofloxacin- bacterial target molecule complex 2D graph**: Ligand color show blue. (a-b) Ciprofloxacin – Bmr complex 2D graph. (c-d) Ciprofloxacin – PBP-1 complex. (e-f) Ciprofloxacin -Dehydratase complex 2D graph. (g-h) Ciprofloxacin - ompC complex 2D graph. (i-j) Ciprofloxacin- Dispersin complex 2D graph.
